# Fibrillar form of α-synuclein-specific scFv antibody inhibits α-synuclein seeds induced aggregation and toxicity

**DOI:** 10.1038/s41598-020-65035-8

**Published:** 2020-05-18

**Authors:** Vijay Gupta, Safa Salim, Issam Hmila, Nishant N. Vaikath, Indulekha P. Sudhakaran, Simona S. Ghanem, Nour K. Majbour, Sara A. Abdulla, Mohamed M. Emara, Houari B. Abdesselem, Tamas Lukacsovich, Daniel Erskine, Omar M. A. El-Agnaf

**Affiliations:** 1 Neurological Disorder Research Center, Qatar Biomedical Research Institute (QBRI), Hamad Bin Khalifa University (HBKU), Qatar Foundation, Doha, Qatar; 2 Translational and Clinical Research Institute, Newcastle University, Newcastle upon Tyne, United Kingdom; 3 Brain Research Institute, University of Zürich, Zürich, Switzerland; 4 Basic Medical Sciences Department, College of Medicine, QU Health, Qatar University, Doha, Qatar

**Keywords:** Parkinson's disease, Parkinson's disease

## Abstract

Synucleinopathies including Parkinson’s disease (PD), dementia with Lewy bodies (DLB), and multiple system atrophy (MSA) are characterized by pathological accumulation of α-synuclein (α-syn). Amongst the various approaches attempting to tackle the pathological features of synucleinopathies, antibody-based immunotherapy holds much promise. However, the large size of antibodies and corresponding difficulty in crossing the blood-brain barrier has limited development in this area. To overcome this issue, we engineered single-chain variable fragments (scFvs) against fibrillar α-syn, a putative disease-relevant form of α-syn. The purified scFvs showed specific activity towards α-syn fibrils and oligomers in comparison to monomers and recognized intracellular inclusions in human *post-mortem* brain tissue of Lewy body disease cases, but not aged controls. *In vitro* studies indicated scFvs inhibit the seeding of α-syn aggregation in a time-dependent manner, decreased α-syn seed-induced toxicity in a cell model of PD, and reduced the production of insoluble α-syn phosphorylated at Ser-129 (pS129-α-syn). These results suggest that our α-syn fibril-specific scFvs recognize α-syn pathology and can inhibit the aggregation of α-syn *in vitro* and prevent seeding-dependent toxicity. Therefore, the scFvs described here have considerable potential to be utilized towards immunotherapy in synucleinopathies and may also have applications in *ante-mortem* imaging modalities.

## Introduction

Synucleinopathies are a group of neurodegenerative disorders comprising Parkinson’s disease (PD), dementia with Lewy bodies (DLB), and multiple system atrophy (MSA)^1^. PD is clinically characterized by motor symptoms of bradykinesia, unsteady gait and resting tremor that precedes cognitive impairment whilst, in contrast, DLB manifests as a cognitive disorder that often leads to motor features^2^. However, despite differences in the sequence of clinical symptomatology, PD and DLB are both neuropathologically characterized by accumulations of the protein α-synuclein (α-syn) in vulnerable neurons as Lewy bodies (LBs) and in neuronal processes as Lewy neurites^3^. MSA is characterized by α-syn aggregates in oligodendroglia as Papp-Lantos bodies/glial cytoplasmic inclusions^4^, though neuronal accumulations of α-syn are also observed^5^.

Several lines of evidence indicate that α-syn aggregation is a critical pathogenic event in the natural history of Lewy body diseases. α-Syn is the major protein component of LBs, various point mutations and/or multiplications in the α-syn gene have been described in familial PD, exogenous expression of α-syn in *Drosophila* and transgenic mice induce the formation of PD-like pathological phenotypes and behavior, and down-regulation of the α-syn protein reduces risk of developing PD^6–13^. Although α-syn is implicated in PD risk, it is also thought to have important neuronal functions as it is a relatively abundant protein, comprising 0.5–1% of the total protein in soluble cytosolic brain fractions^14,15^.

Although α-syn is a soluble, monomeric, unfolded protein, it can convert to various conformations such as helically folded tetramers resisting aggregation or oligomers, small aggregates, protofibrils or irreversible insoluble amyloid fibrils based on the cellular environmental stimuli and genetic factors^16–18^. Formation of α-syn fibrils is a multistep reversible heterogeneous reaction that can be initiated by conversion of native soluble α-helix rich protein into the pathogenic β-sheet structures, utilizing small oligomers or fibrils as seeds for propagation^19^. Multiple lines of evidence suggest that the oligomeric or fibrillar form of α-syn mediates toxicity causing neurodegeneration and neuronal cell-death leading to PD and other synucleinopathies^20–22^. Therefore, molecules that can detect, bind and inhibit the toxic oligomeric and fibrillar species of α-syn can be used as diagnostic and therapeutic tools for synucleinopathies.

Strategies involving active and passive immunizations have been shown to ameliorate the symptoms of synucleinopathies using α-syn antibodies in animal models of PD, DLB and MSA. However, the large size of monoclonal antibodies, limiting their ability to cross the blood-brain barrier (BBB), are major limitations to this approach^23^. One of the applications of recombinant DNA technology is the generation of phage or yeast surface display antibody libraries consisting of variable domains of heavy chain (V_H_)and light chain (V_L_) fragment in multiple permutation-combinations that can be used to screen for functional single-chain variable fragment (scFv) antibodies against any target antigen. Alternatively, already characterized functional monoclonal antibodies can be sequenced and V_H_ and V_L_ sequences responsible for antigen binding can be identified and cloned to synthesize scFv gene.

ScFv is a small antigen-binding molecule, which consists of the V_H_ and V_L_ regions linked by a short flexible linker, generally (Gly_4_Ser)_3_. Additionally, scFv can be genetically engineered to include chemical tracers or a cell-penetrating peptide, thus raising the possibility of using these tools for biomarkers or therapeutics. Therefore, scFv fragments preserve the antigen-binding capacity, affinity and specificity of antibodies but with lower mass, better penetration in tissues, shorter half-lives and faster clearance. Moreover, as scFv lack the tail Fc region of antibodies that interacts with the immune system, these fragments are less likely to initiate a potentially deleterious immune response than antibodies^24–29^.

We previously described a conformation-specific anti-α-syn monoclonal antibody (Syn-F2) that specifically recognizes α-syn fibrils^30,31^. For our current study, using the sequence from Syn-F2, we produced scFv antibodies with and without a cell-penetrating peptide (CPP) using *E. coli* expression system. We analyzed scFvs binding to α-syn fibrils and oligomers and their effect on α-syn seeding induced aggregation and toxicity. We found scFvs to be α-syn fibril and oligomer specific *in vitro* and they labelled intracellular aggregates in *post-mortem* tissue of cases with Lewy body pathology. Furthermore, they were capable of inhibiting the seeding of α-syn aggregation in an *in vitro* assay, whilst reducing the toxicity caused by α-syn seeds in cell model. scFvs also blocked the aggregation of α-syn as detected by decreased insoluble α-syn phosphorylated at Ser-129 (pS129-α-syn) in another cell model. These results suggest that α-syn pathology-specific scFvs are able to specifically bind α-syn fibrils and oligomers and inhibit the seeding of α-syn aggregation that might lead to the pathogenesis of PD, DLB and MSA.

## Results

### Generation of α-syn fibril-specific scFvs

**Figure 1:**
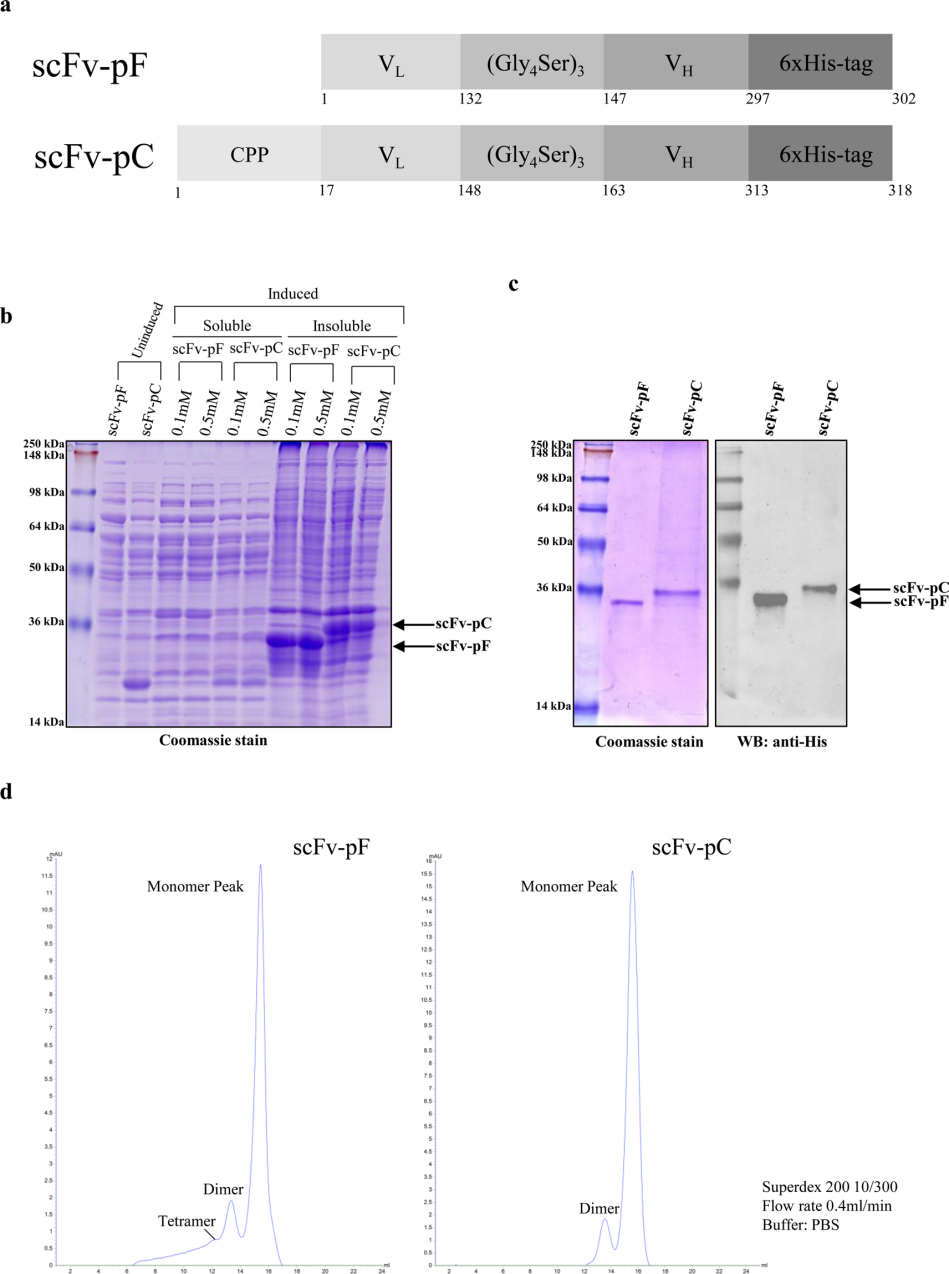
Schematic representation of the scFv-pF and scFv-pC fusion proteins, their expression and purification. (**a**) Light chain (V_L_) and heavy chain (V_H_) variable domains were linked by a (Gly_4_Ser)_3_ linker with a C-terminal 6xHis-tag. Amino acid number starting for each domain is marked. scFv-pC consisted of an N-terminal CPP sequence. (**b**) SDS-PAGE analysis of expression of scFv-pF and -pC in soluble/insoluble fractions of BL21(DE3) cells upon overnight incubation at 25 °C, induced by different IPTG concentrations. scFv-pF and -pC are indicated by an *arrow*. (**c**) SDS-PAGE (left) and western blot (right) analysis of His-trap purified and dialysed scFv-pF and -pC. Molecular masses (kDa) of protein standards are indicated on the left. (**d**) Size exclusion chromatography showing the monomeric scFv-pF and -pC, proteins as major fractions.

The single-chain variable fragment (scFv) antibodies, scFv-pF and scFv-pC were generated by cloning DNA sequences of V_L_ and V_H_ domains from Syn-F2 antibody sequence in pET-28a expression vector. The variable domains were connected by a (Gly_4_Ser)_3_ linker sequence and a 6x-His tag for convenient purification and detection was added at the C-terminus. scFv-pC had a cell-penetrating peptide (CPP)-RQIKIWFQNRRMKWKK at its N-terminus to facilitate intracellular delivery (Fig. 1a). Protein expression vectors for scFv-pF and -pC were transformed into *E. coli* BL21 (DE3) cells and induction profiling was performed at various IPTG concentrations. Surprisingly, higher expression of scFv-pF and -pC was observed in the insoluble fractions in comparison to soluble fractions. Efforts to optimize and enhance soluble expression of scFvs utilizing various conditions such as lower growth temperatures of 16 °C, 20 °C and 25 °C, different IPTG concentrations (from 0.01 mM to 1 mM), addition of various chaotropic agents e.g. sorbitol, ethanol, and L-arginine, were largely unsuccessful. Therefore, we decided to purify the scFvs from the insoluble fractions containing *E. coli* inclusion bodies (IBs). Expression of scFvs in the insoluble fractions was found to be highest upon induction with 0.1 mM IPTG overnight at 25 °C (Fig. 1b). Traditional methods to dissolve IBs involve using high concentrations of chaotropic agents such as urea (8 M) or guanidine chloride (6 M) which lead to complete denaturation of active protein, rendering it unable to display antigen binding properties. High amount of urea or guanidine chloride is further removed from denatured proteins using extensive slow or sequential dialysis protocols which simultaneously refold inactive proteins to gain activity back. A mild solubilization buffer containing 50 mM Tris-HCl, and 2 M urea at pH 12.5 was chosen to solubilize the IBs^32^. ScFv fragments were purified using FPLC according to the protocol described in methods and urea solubilization method gave a yield of ~50% as shown in (Suppl. Fig. S1a). Western blotting using anti-His antibody confirmed the presence of full-length scFv fragment after purification (Fig. 1c) and size exclusion chromatography showed that most of the purified protein is in the monomeric state with some dimers and tetramers which were separated and monomeric proteins were used for further experiments (Fig. 1d). Protein standards of known molecular weights were run to determine the corresponding size and elution profile (Suppl. Fig. S1b).

### scFv-pF and -pC specifically recognize α-syn fibrils and oligomers

**Figure 2:**
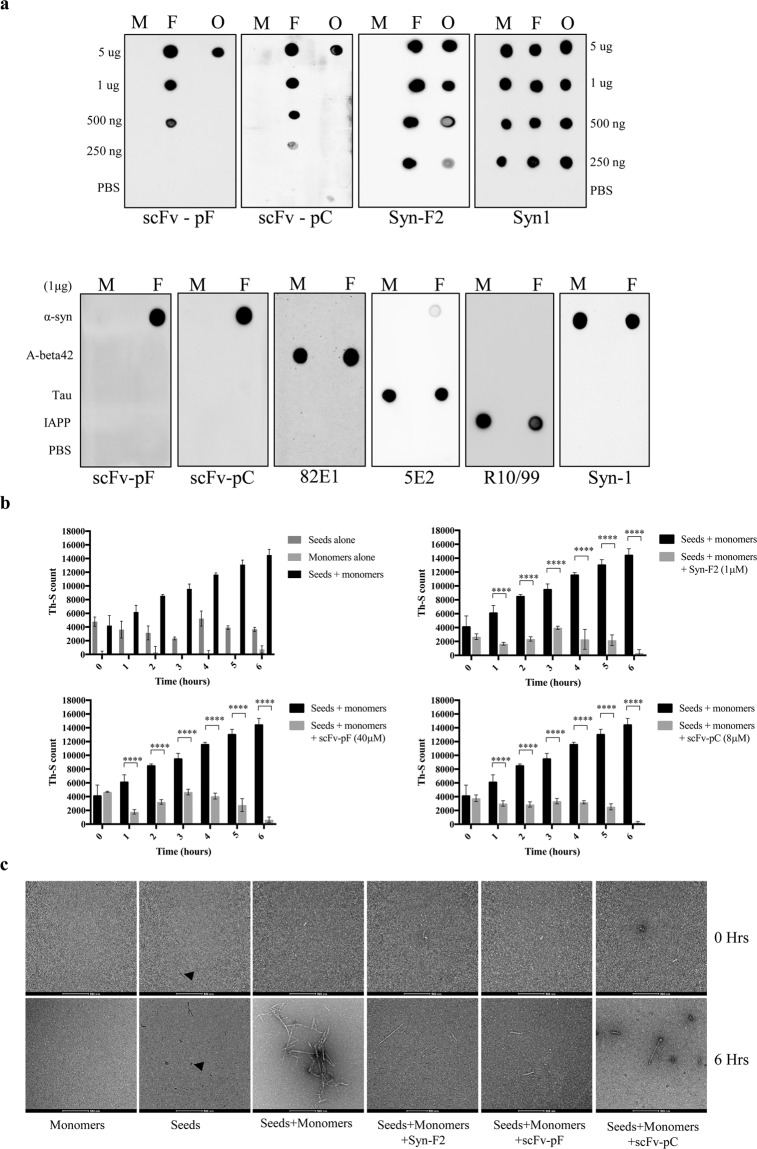
Characterization of purified scFv-pF and scFv-pC. (**a**) Dot blot showing specific binding of scFv-pF and -pC to fibrillar and oligomeric forms of α-syn. Indicated amounts of full-length α-syn monomers (M), fibrils (F) or oligomers (O) (upper panel) and 1 μg of A-beta 42, Tau, IAPP (bottom panel) were spotted onto a nitrocellulose membrane. The membranes were probed with scFv-pF/-pC and Syn-F2 for α-syn, 82E1 for A-beta 42, 5E2 for Tau, R10/99 for IAPP antibodies, and Syn-1 for full-length α-syn. (**b**) *In vitro* seeding of α-syn aggregation assay showing inhibition by Syn-F2, scFv-pF and -pC. α-Syn monomers (25 µM) were seeded with 1 µM α-syn seeds, which were incubated in the presence or absence of Syn-F2 (1 µM), scFv-pF (40 µM) and scFv-pC (8 µM) for 6 hours with continuous shaking at 37 °C. The extent of fibrillation was estimated by the Th-S fluorescence assay at indicated time-points. The assay was performed in triplicate (average of triplicate measurements ± standard deviations). Statistical analysis was performed using two-way ANOVA with Sidak’s multiple comparison test. (****p < 0.0001). (**c**) Electron microscopy images of negatively stained samples collected at time 0 and 6 hours from experiment in (**b**) show that mature amyloid fibrils (300–700 nm long) are formed in seeds and monomer incubated samples at 6 hours time point which is inhibited by Syn-F2, scFv-pF and scFv-pC antibodies. Arrowhead indicate presence of seeds at the time-point 0 and 6 hours samples. Magnification 28500x. Scale bar = 500 nm.

To determine the activity and specificity of the purified scFvs we employed different biochemical techniques. First, we used dot blotting to assess the binding specificity of the purified scFvs for different forms of α-syn. For this purpose, we spotted purified α-syn monomers, dopamine-induced oligomers (DA-oligomers) and fibrils on nitrocellulose membranes and probed using the scFvs. It was observed that the scFvs bound with higher specificity to α-syn fibrils and DA-oligomers in comparison to α-syn monomers (Fig. 2a, top panel). scFv-pC had slightly better binding than scFv-pF since it showed binding with 250 ng α-syn fibrils compared to scFv-pF which showed binding with only up to 500 ng α-syn fibrils (Fig. 2a, top panel). Syn-F2 antibody was used as a positive control and Syn-1 antibody was used in dot blots to determine the equal loading of α-syn antigens (Fig. 2a, top panel)^30^. After determining the specificity of scFvs for α-syn fibrils and oligomers, we wanted to examine whether this binding is specific for α-syn fibrils or the scFvs can non-specifically bind to any other type of amyloid fibrils as well. Monomeric and fibrillar forms of various amyloidogenic-type proteins such as A-beta 42, tau, and islet amyloid polypeptide (IAPP) were coated and dot blots were performed. scFv-pF and -pC showed binding only with α-syn fibrils and not with A-beta 42, tau or IAPP proteins, confirming its specificity towards α-syn fibrils (Fig. 2a, bottom panel).

In order to further evaluate the specificity of scFvs for α-syn fibrils, we performed indirect ELISA using α-syn monomers and fibrils. scFvs showed high binding to α-syn fibrils compared to monomers replicating the data observed in dot blotting (Suppl. Fig. S2a) whereas, the control antibody, Syn-1 showed equal reactivity to both α-syn monomers and fibrils (Suppl. Fig. S2b). These results showed that purified scFv-pF and -pC had correctly folded antigen-binding sites that efficiently bound the α-syn fibrils.

### **scFvs inhibit seeding of α-syn aggregation in*in vitro*assay**

Previous work had shown that amyloid fibrils are formed in a nucleation-dependent polymerization fashion^33^. Based on this model, there are three phases: first, a nucleation or lag phase wherein soluble species are generated via nucleation of oligomeric species; second, a polymerization or growth phase in which oligomeric species polymerize giving rise to fibrils; and a final equilibrium phase where it reaches plateau^34^. It has been demonstrated that small aggregates or seeds accelerate the nucleation phase of amyloid formation by a process called “seeding” both *in vitro* and *in vivo*^35–38^. According to established protocols, to generate α-syn “seeds”, mature α-syn fibrils were sonicated to fragment into smaller short fibrillar pieces (Suppl. Fig. S2c,d). The seeds were added to α-syn monomers and the mixture was incubated to allow the aggregation process to take place. Incubation of seeds with α-syn monomers speeded up the fibril formation which was demonstrated by enhanced Th-S binding. The quantity of α-syn fibrils formed upon addition of seeds in the six hours duration is equivalent to α-syn fibrils formed in 72 hours without addition of seeds^39,40^.

Since the scFvs and Syn-F2 showed specific binding to α-syn fibrils, we evaluated whether they can prevent the seeding of α-syn monomers to fibrils. To determine the effect of the scFvs on the seeding of α-syn monomers, α-syn seeds (1 μM-minimum amount required for efficient seeding) were incubated either alone or in combination with Syn-F2 (1 μM), scFv-pF (40 μM) or scFv-pC (8 μM) for 1 hour at 37 °C with continuous shaking, followed by incubation with 25 μM α-syn monomers. The reaction was allowed to occur for six hours at 37 °C and samples were collected at every hour. α-Syn monomers or seeds alone had no appreciable change in Th-S counts at all time-points however samples having both α-syn monomers and seeds showed significant increase in fibril formation as indicated by Th-S counts (Fig. 2b) and further confirmed by electron microscopy data (Fig. 2c), with increasing time-points. As shown in Fig. 2b, scFv-pF and scFv-pC inhibited the seeded fibrillation of α-syn monomers significantly (p-values < 0.001) similar to Syn-F2, as evident by the low count of Th-S binding in the presence of the scFvs. Similar inhibitory results were obtained at 24 hours time-point by scFv-pF/-pC and Syn-F2 (Suppl. Fig. S2f).

Moreover, scFvs or Syn-F2 incubated alone without seeds did not have any effect on the aggregation of α-syn monomers. α-Syn monomers had negligible Th-S counts indicating the absence of any fibrillary structures, which did not increase by Syn-F2 or scFv-pF/-pC addition (Suppl. Fig. S3a). More importantly, α-syn seeds alone, in the absence of α-syn monomers, incubated with Syn-F2, scFv-pF or scFv-pC had only some basal counts of Th-S, which was not enhanced by Syn-F2 or scFv-pF/-pC co-incubation (Suppl. Fig. S3b). The concentration required for the scFvs to inhibit the seeded aggregation of α-syn monomers is higher compared to Syn-F2. The lower affinity of these scFvs compared to Syn-F2 might possibly be the avidity effect due to the presence of only one antigen binding domain. Western blotting using samples from *in vitro* seeding of α-syn aggregation assay (Fig. 2b) showed inhibition of high molecular weight aggregate bands by Syn-F2, scFv-pF and -pC co-incubation compared to seeds and monomer alone at 6 hours time-point (Suppl. Fig. S2e).

### CPP allows the transport of scFv across live cell membrane

The key challenge towards antibody-based therapy is effective delivery of the therapeutic bioactive molecule at the site of treatment i.e. the brain in the case of neurodegenerative diseases. Addition of cell permeable peptide molecules has been shown to facilitate delivery of target molecules to intracellular locations as well as to aid in crossing the blood-brain barrier (BBB)^41,42^. Recently, cell-penetrating peptide (CPP) has also been shown to deliver cargo molecules into a variety of cell systems and across the BBB^41^. We decided to use a 16-amino acid long CPP obtained from the third helix of the *Antennapedia* homeodomain from *Drosophila* in our scFv coding sequence to enable it to cross cell membranes^29,42^.

**Figure 3:**
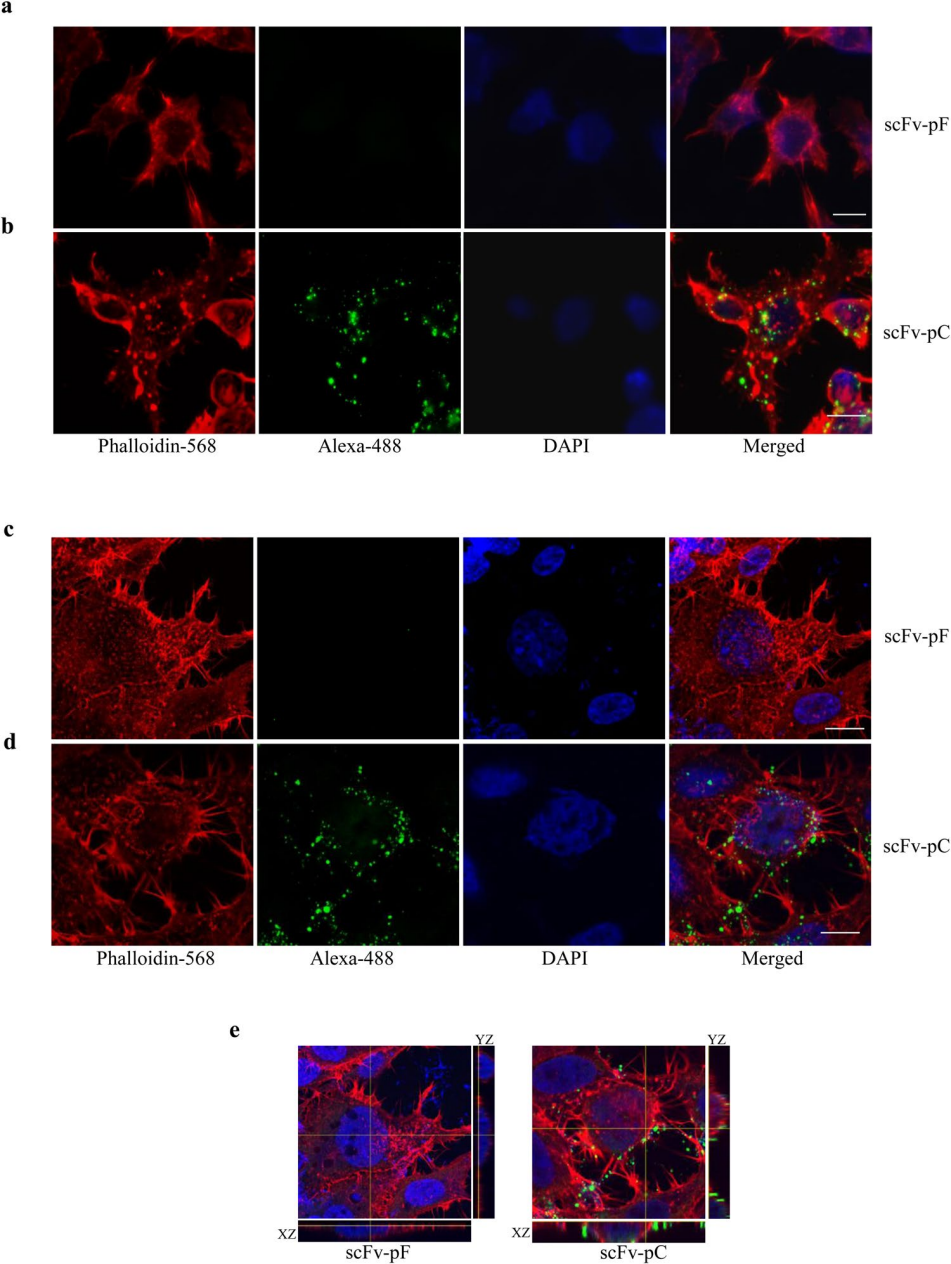
Intracellular delivery of scFv by CPP in SH-SY5Y and HEK293T cells. SH-SY5Y (**a,b**) or HEK293T (**c,d,e**) cells incubated with scFv-pF and -pC for 120 min in Opti-MEM were fixed and stained using anti-His antibody and Phalloidin-594 and imaged using confocal microscope. Serial optical sections in the *Z*-axis of the cell, collected at 1 μm intervals with 63x oil immersion objective lense (NA 1.4) were projected and observed in a total thickness of 10 μm by using LSM 780 (version 3.2) software. scFv-pF (a or c) is not showing any specific staining indicating no intracellular delivery due to the lack of any cell penetrating peptide. scFv-pC (**b,d**) is showing good specific staining around the cells and inside cells indicating its binding to the cell membrane and possibly intracellular delivery due to the presence of CPP-peptide. (**e**) Merged confocal image with the orthogonal projection of scFv-pF (left) and scFv-pC (right) localization along with XZ and YZ views. Scale bars = 10 μm.

To detect if there was any favorable effect of addition of CPP in scFv sequence towards cell delivery, scFv without any peptide (scFv-pF) or scFv fused with CPP (scFv-pC) were incubated with human neuroblastoma SH-SY5Y cells or HEK293T cells in Opti-MEM media for 120 min at 37 °C. Opti-MEM media containing scFvs was removed, cells were extensively washed, fixed and stained using anti-His antibody to visualize scFv proteins using confocal microscope. Serial optical sections in the *Z*-axis of the cell, collected at 1 μm intervals (total thickness of 10 μm) were projected as a flat single image and observed. scFv-pC showed vesicular and membranous pattern across multiple cells indicating binding to cell membranes as well as intracellular localization (Fig. 3b,d). On the other hand, scFv-pF had undetectable signals by immunofluorescence microscopy (Fig. 3a,c), confirming that scFv-pF is unable to cross the cell membrane by itself at this time-point. Further analysis of individual z planes using orthogonal projection along with XZ and YZ views confirmed internal localization of scFv-pC (Fig. 3e, right) but not for scFv-pF (Fig. 3e, left). Phalloidin-594 was used as a control to mark areas of cytoskeletal structure.

### scFvs blocks α-syn aggregates-induced toxicity

Amyloid fibrillation is known to generate oligomeric/fibrillar intermediates causing cell death^43–45^. To analyze the ability of scFv to block α-syn-mediated cellular toxicity, an MTT assay was performed using a SH-SY5Y cell model of PD as previously established^45,46^. In living functional cells, the tetrazolium salt, MTT, gets reduced to formazan by mitochondrial dehydrogenase. Incubation of SH-SY5Y cells with α-syn seeds (2 μM) followed by α-syn monomers (10 μM) leads to the formation of toxic α-syn fibrils in a seeding model, thus reducing cell viability. Previous report has suggested that α-syn seeds-dependent toxicity is enhanced greatly by adding α-syn monomers^45^.

**Figure 4:**
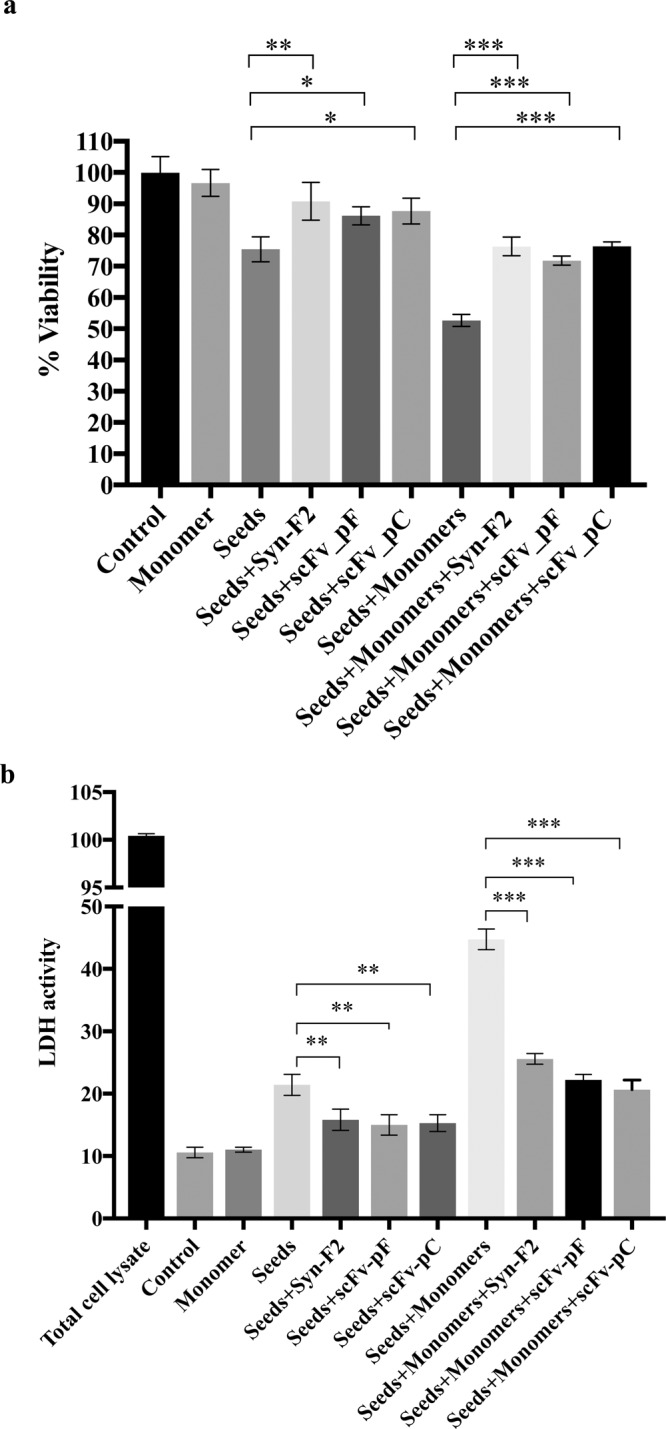
scFv-pF and scFv-pC reduce the toxicity caused by α-syn seeds in SH-SY5Y cell model of PD. The viability of SH-SY5Y cells was evaluated using MTT assay (**a**) or LDH activity assay (**b**). 2 µM α-syn seeds were incubated with 0.25 µM Syn-F2, 0.25 µM scFv-pF, or 0.25 µM scFv-pC. After incubation, 10 µM α-syn monomers were then added in Opti-MEM for 48 hours prior to MTT addition. For MTT assay, the results are expressed as percentages of the average of the control (untreated cells). For LDH assay (**b**) 50 μl media was removed before the addition of MTT reagent and released LDH activity was measured. The absorbance was measured and percentage of the viable cells or LDH activity was plotted (average of 3 wells ± standard deviation). Statistical analysis was performed using one-way ANOVA with Dunnet’s multiple comparison test. (***p < 0.001; **p < 0.01; *p < 0.05).

SH-SY5Y cells were treated with α-syn seeds in the presence or absence of Syn-F2, scFv-pF or scFv-pC in two identical sets with or without α-syn monomer addition. As shown in Fig. 4a, consistent with the role of the scFvs in inhibiting α-syn seeds-mediated aggregation (Fig. 2b), they also reduced the toxicity caused by α-syn seeds, giving rise to a significantly higher number of viable cells as detected by increased formazon absorbance (Fig. 4a, p-values  < (0.05–0.001)). Syn-F2 antibody was used as a positive control and it also increased cell viability showing its inhibitory role in α-syn seeds- mediated toxicity (Fig. 4a). Since scFvs and Syn-F2 inhibited the toxicity caused by α-syn seeds with monomer addition, it demonstrated that seeded aggregation is blocked. However, scFvs and Syn-F2 co-incubation with α-syn seeds without monomer addition also reduced the toxicity indicating that these antibodies inhibits its internalization as well as further seeded aggregation. These results were also confirmed by measuring the activity of released LDH in the media (Fig. 4b). In any case, aggregation is not completely inhibited by scFvs and Syn-F2 perhaps due to the secondary mechanisms employed by α-syn seeds and monomers once inside the cells to cause toxicity, such as interactions with mitochondrial membranes^47^.

### scFvs reduce insoluble pSer129 α-syn level in cell model

α-Syn has been found to be modified by a variety of post-translational modifications such as phosphorylation, acetylation, nitration, glycosylation, SUMO-ylation and ubiquitination^48–55^. However, the exact role of these modifications in α-syn functional regulation or with respect to the role of α-syn in PD pathogenesis is not clear. pS129-α-syn is the most abundant post-translational modification shown in LBs^48^. ~90% of aggregated α-syn in LBs is pS129-α-syn compared to only ~4% α-syn in normal cellular conditions^56^. Such abundance of pS129-α-syn implicates an important role of phosphorylation in α-syn aggregation and pathogenesis of PD. Recent reports have shown that pS129-α-syn may play a critical role in speeding PD pathogenesis by decreasing affinity of pS129-α-syn to membranes, thereby enhancing its aggregation^57^.

**Figure 5:**
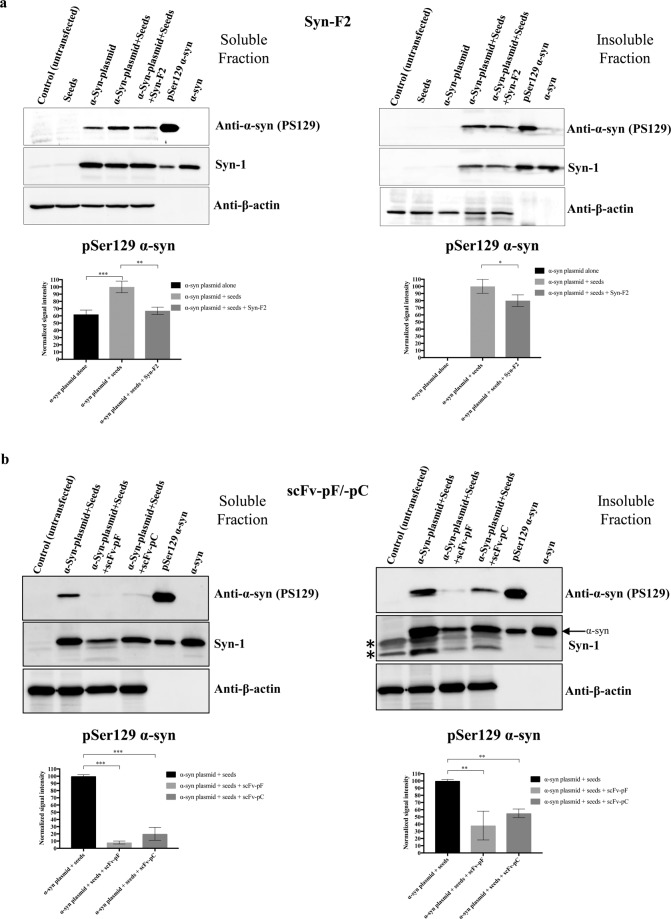
Syn-F2, scFv-pF and scFv-pC decrease insoluble α-syn level in HEK293T cell model of PD. HEK293T cells were transfected with wild type α-syn plasmid using lipofectamine 3000 reagent. One group of α-syn plasmid expressing HEK293T cells was similarly transfected again with 0.2 µM α-syn seeds the following day and then incubated with 0.25 µM Syn-F2 (panel a), 0.25 µM scFv-pF (panel b), or 0.25 µM scFv-pC (panel b) for next 48 hours. Triton X-100 soluble (soluble fraction) and SDS-soluble (insoluble fraction) protein extracts were prepared, and presence of pSer129 α-syn and total α-syn was detected using anti-phospho-α-syn and Syn-1 (stripped blot) antibodies by western blotting sequentially. In (**b**) ** denotes non-specific bands in insoluble fraction and specific band of α-syn is marked by arrow. β-Actin was used as a loading control and detected by anti-β-actin antibody (stripped blot). Images from the same blot was cropped to show anti-phospho-α-syn, Syn-1 and β-actin separately. Full blot scans can be found in supplementary data file. The experiment was repeated three times and intensity of pSer129 α-syn bands was normalized to β-actin and Syn-1 and plotted. Statistical analysis was performed using one-way ANOVA with Dunnet’s multiple comparison test. (***p < 0.001; **p < 0.01; *p < 0.05).

Previous work in our laboratory as well as by other groups has shown that α-syn seeds when incubated with α-syn monomers lead to the formation of fibrils and enhance aggregation^45^. We developed HEK293T cell model wherein exogenous α-syn expression recapitulates the aggregation induction upon α-syn seeds addition. Immunocytochemistry data (Suppl. Fig. S6) showed that α-syn expression in presence of seeds lead to the formation of insoluble aggregates as detected by pSer129 staining. Effect of antibodies on α-syn expression, aggregation and phosphorylation can be tested after seeds internalization by adding them in cell-culture media. This aggregated α-syn is detected by enhanced levels of insoluble pSer129 α-syn. α-Syn exogenous wild-type plasmid expression alone showed the presence of a basal level of phosphorylated α-syn in the soluble fraction (due to the overexpression of α-syn); however, there was no phosphorylated α-syn in the insoluble fraction (Fig. 5a). Addition of α-syn seeds alone did not show any phosphorylated or non-phosphorylated α-syn protein (Fig. 5a). We asked whether Syn-F2 or scFvs can decrease insoluble pSer129 α-syn in this cell model and observed that both scFvs significantly reduce levels of pSer129 α-syn in the soluble and insoluble fractions (Fig. 5b, p < 0.001). On the other hand Syn-F2 had marginal effect in reducing the level of pSer129 α-syn in the soluble and insoluble fractions (Fig. 5a, p < 0.05). We also verified the presence of total α-syn by western blotting using pan-α-syn antibody, Syn-1. Quantification of the western blot results from three independent experiments showed that there was a significant decrease in insoluble pS129-α-syn (normalized to β-Actin and Syn-1 protein levels) following addition of scFv-pF and scFv-pC (Fig. 5b, p < 0.01, Suppl Fig S5c,d). Quantification of Syn-1 densitometry showed that scFvs treatment also reduced α-syn protein level which might be due to binding of scFvs with the early intermediate of α-syn fibril formation and those being cleared by cellular machinery (Suppl. Fig. S5a,b).

Based on the data obtained in Fig. 5, scFv-pF as well as scFv-pC both inhibited pSer129 α-syn levels drastically compared to Syn-F2. We speculate that it might be that scFvs with their small size can also bind aggregates that IgG cannot recognize. We decided to gain more knowledge about the mechanism of scFvs in reducing levels of pSer129 α-syn, whether it is due to engagement of scFvs with α-syn seeds exclusively extracellular or is there any intracellular contribution as well. We performed a time-course experiment wherein we incubated scFv proteins (scFv-pF/-pC) with SH-SY5Y cells for 30 min, 2 hours, 6 hours and 24 hours, fixed and stained using anti-His antibody and scFv proteins were visualized using wide-field microscope (Suppl. Fig. S4). scFv-pC showed efficient binding to cell membranes and intracellular delivery at all time-points from 30 minutes to 24 hours whereas scFv-pF did not show any specific staining pattern at initial time-points of 30 min and 2 hours, however at later time points of 6 hours and 24 hours it started showing cell membrane binding (Suppl. Fig. S4). Since scFvs are smaller molecules (~35k Da) compared to full-length IgG molecules (150 kDa), it is highly probable that scFvs can bind to cell membrane, get internalized and, once inside the cells, can mediate effects based on its antibody-like properties.

### scFvs detect intracellular inclusions in human brain tissues from PD and DLB patients

**Figure 6:**
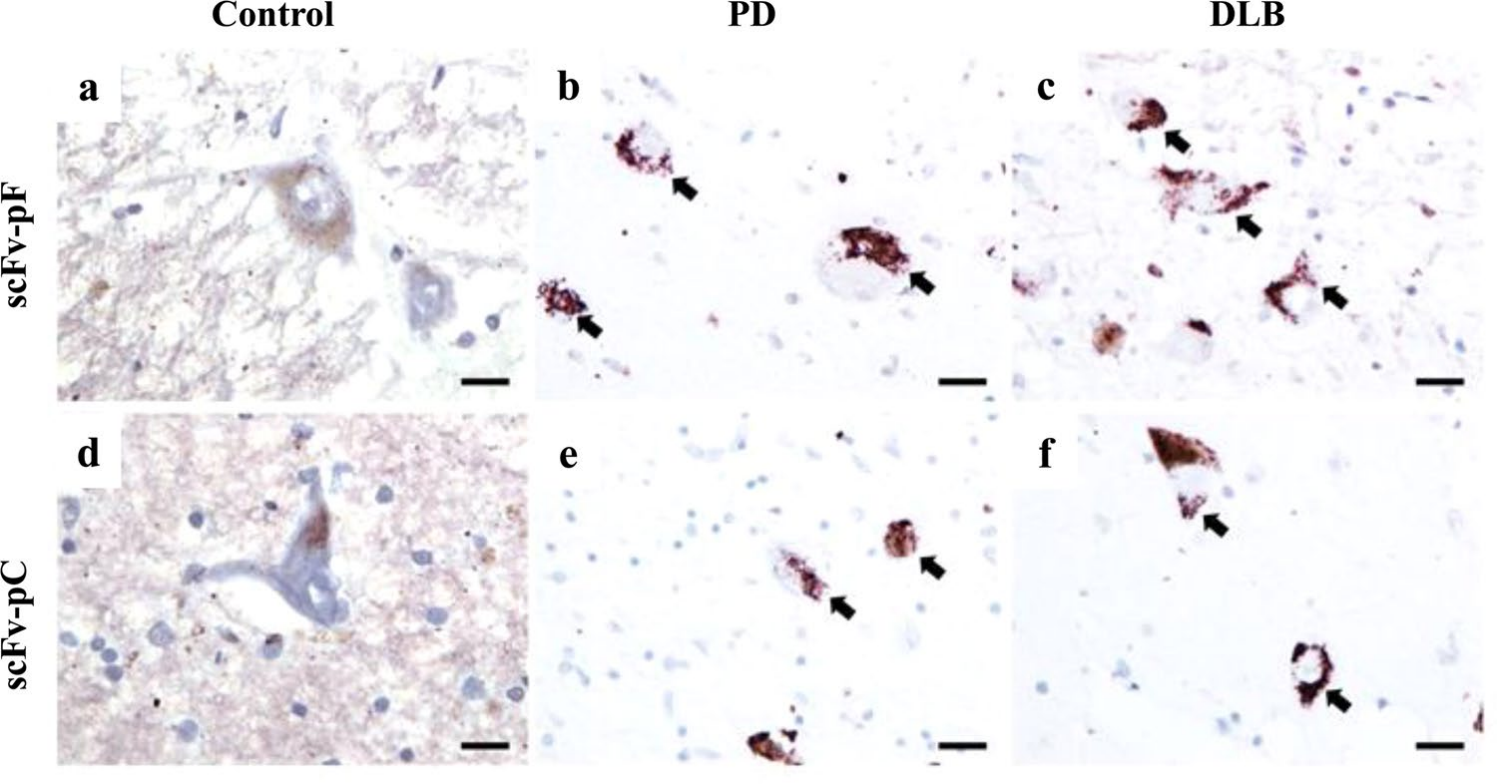
Immunohistochemistry data using human post-mortem brain tissues shows disease specific cytoplasmic granules detected by scFv-pF and scFv-pC. Staining with scFv-pF and scFv-pC in substantia nigra demonstrated an absence of staining in nigral neurons (**a,d**), whilst PD (**b,e**) and DLB (**c,f**) had granular intracytoplasmic deposits that were immunoreactive for both scFvs. Scale bars = 20 µm.

To confirm whether scFvs recognized relevant protein aggregates in human brain tissue, we used formalin-fixed paraffin-embedded *post-mortem* brain tissue from individuals with clinical and neuropathological diagnoses of PD (N = 1) and DLB (N = 1), in comparison to aged controls without α-syn pathology (N = 2). Staining with scFvs using immunohistochemistry in substantia nigra demonstrated faint background staining in control cases that could be native α-syn whilst diffuse granular deposits were observed in nigral neurons in PD and DLB cases that were not observed within control cases (Fig. 6).

## Discussion

Current therapies for the synucleinopathies are symptomatic in nature and lack any significant disease-modifying effect. Therefore, the development of novel and effective therapies to arrest or ameliorate the progression of synucleinopathies is a pressing therapeutic priority. As the aggregation of α-syn is thought to be a critical pathogenic event in the natural history of synucleinopathies^46,58,59^, many candidate therapeutics in current development aim to target and inhibit this process.

Naturally occurring antibodies, C-terminal targeting antibodies and conformation-specific antibodies against α-syn aggregates ameliorate the disease-related phenotype of mouse models, with some already in different stages of human clinical trials^31,60,61^. However, a major limitation of antibody-based therapeutics is the large size of antibodies, limiting permeability to the blood-brain barrier, and often necessitating infusion directly onto the brain, an invasive procedure with increased risk of cerebral infection^62^.

With the advent of recombinant DNA technology, scFv can be designed by fusing variable regions of heavy and light chain with a polypeptide linker using already characterized monoclonal antibody derived sequences^23^. Critically, scFvs are considerably smaller than antibodies, enabling improved permeability to the blood-brain barrier, enabling ready application at peripheral sites with improved efficiency of passage to the central nervous system in therapeutically meaningful doses. scFvs are believed to show similar binding affinity and specificity compared to antibodies due to maintenance of similar antigenic binding sequence as the parent antibody. Moreover, scFv sequence can be modified to enhance affinity, avidity or intracellular targeting^23,42,63^. By screening various phage and yeast surface display libraries, some α-syn conformation-specific scFvs have been developed, and shown to affect different steps of α-syn aggregate formation and/or ameliorate α-syn neuropathology in different *in vitro* and *in vivo* model systems^25–27,29,64–66^. However, to the best of our knowledge, the scFvs reported in this study are the first of its kind based on a well-characterized conformation-specific monoclonal antibody, the Syn-F2 sequence^30^.

Our findings with our novel scFvs demonstrate, for the first time, that scFvs can employ well-validated conformation-specific sequences from antibodies, and use these for potential therapeutic applications. Our scFvs recognize α-syn fibrils specifically, and identify disease-specific intracellular accumulations in *post-mortem* brain tissue that are not observed in aged control cases. Finally, scFvs rescue cells from α-syn-mediated cellular aggregation and toxicity *in vitro*. Taken together, our findings suggest scFvs may be a viable therapeutic option for the treatment of synucleinopathies. By linking to tracer compounds scFvs can also be developed as attractive target to be used in *in vivo* imaging applications as diagnostic tool towards synucleinopathies^23,63,67,68^. Future studies should aim to analyze the effect of the scFvs in other *in vivo* and animal models of synucleinopathies, which will provide important insights into safety and tolerability in intact organisms, in addition to the α-syn aggregation blocking properties of this class of scFv.

In conclusion, we have generated α-syn pathology-specific scFvs that can modulate α-syn aggregation indicating its therapeutic and diagnostic use in synucleinopathies. Given that α-syn aggregation is the neuropathological hallmark of Lewy body diseases and is thought to be central to the disease process, scFvs targeting disease-relevant conformations have considerable potential in the search for novel therapeutics to attenuate α-syn aggregation.

## Materials and Methods

### **Cloning*E.coli*expression constructs**

Sequences of the V_L_ and V_H_ regions from Syn-F2 were obtained using GenScript antibody sequencing service, and used to generate scFv construct in pET22b(+)vector. The construct consisted of the V_L_ and V_H_ regions linked by a (Gly_4_Ser)_3_ linker and a C-terminal 6x-His tag for convenient purification. For cloning new-constructs, forward (32 bp) and reverse primers (30 bp) were designed corresponding to the V_L_ and V_H_ sequence of Syn-F2 respectively. To add cell-penetrating peptide (CPP) to the scFv expression vector, another forward primer was designed containing the nucleotide sequence coding for CPP-amino acids - RQIKIWFQNRRMKWKK at the starting of the forward primer. Using the pET22b(+) vector containing the coding sequence for our scFv as a template and the newly designed forward and reverse primers, PCR was performed to amplify the scFv total coding sequence. The identity of the final constructs scFv-pC (with CPP) and scFv-pF (without CPP) was confirmed by DNA sequencing (Suppl. Fig. S7).

### Expression and purification of scFvs

The scFv-pF and scFv-pC constructs in pET28a vector were transformed by heat shock into *E.coli* BL21(DE3) bacteria for protein expression. scFv expression was induced by adding 0.1 mM isopropyl β-D-1-thiogalactopyranoside (IPTG), and the culture was grown overnight at 25 °C. The pellet was resuspended in lysis buffer containing 50 mM Tris pH 8.0, 150 mM NaCl, 10% glycerol, 1X protease inhibitor, 1 mM PMSF, 20 U/ml DNAse, and 10 mM MgCl_2_ and sonication cycles of 10 sec upto 5 min with 30 sec on ice were done at 30 amplitude. For purification from inclusion bodies, the pellet was washed 5 times with 50 mM Tris-HCl pH 8.0, 100 mM NaCl, 1% triton X-100, 1 M urea buffer and once with 1X PBS. The pure inclusion body pellet obtained was dissolved in 100 mM Tris-HCl, 2 M urea, pH 12.5 buffer. The supernatant was purified by Ni-NTA affinity chromatography (GE Healthcare) with the addition of linear refolding gradient wherein a linear gradient of 2 M urea to no urea was performed to refold the denatured bound protein via a process called “on-column refolding”. After refolding, the non-specific bound proteins were washed extensively using 40 mM imidazole and our His-tagged protein was eluted using 250 mM imidazole. Overnight dialysis using 1xPBS was performed at 4 °C to remove imidazole from the obtained protein solution. BCA assay was performed to estimate protein concentration and purified protein was frozen in small aliquots at −80 °C.

### Western blot

The samples were boiled with 5X SDS-lamelli sample buffer and separated on 12% SDS-PAGE gel. Proteins were transferred to nitrocellulose membrane and developed using anti-6X-His tag mouse antibody (1:2500, Abcam) followed by incubation with HRP-conjugated IgG goat anti-mouse antibody (Thermo Scientific) at a dilution of 1:10,000 using SuperSignal West-Pico -Chemiluminescent Substrate. All blots were imaged in Bio-Rad ChemiDoc MP imaging system.

### Preparation of α-syn monomers, oligomers, fibrils and seeds

The different forms of α-syn were prepared as described previously^40^. Briefly, for preparation of α-syn fibrils, 25 μM of α-syn samples were aged in PBS for 5 days at 37 °C with continuous shaking at 800 rpm. The formation of α-syn fibrils was monitored by Th-S fluorescence assay. Oligomers were prepared according to previously published protocol^69^. α-Syn seeds were generated by fragmenting mature α-syn fibrils through sonication. To prepare monomers, α-syn was passed through a 100 kDa molecular weight cut-off (MWCO) filter to get rid of high molecular aggregates.

### Dot blot

Nitrocellulose-membrane was spotted with 5 µl/spot of monomeric and aggregated forms of α-syn, A-beta 42, tau and islet amyloid polypeptide (IAPP) proteins. The samples were dried for 30 min followed by blocking in 5% skimmed milk (in PBST). The membranes were incubated overnight at 4 °C with scFv-pF (5 µg/mL) or scFv-pC (5 µg/mL) or respective control antibodies: Syn-1 (for α-syn, BD Biosciences, 50 ng/ml); 82E1 (for A-beta, IBL America, 50 ng/ml); 5E2 (for Tau, kindly provided by Prof. Dominic Walsh, Harvard Medical School, 50 ng/ml); and R10/99 (for IAPP, Santa Cruz Biotechnology 100 ng/ml). Post incubation the blots were developed similar to western blot.

### Indirect enzyme-linked immunosorbent assay (ELISA)

A 96-well clear MaxiSorp plate (Nunc) was coated with 100 ng/100 µL (per well) of α-syn monomers and fibrils in PBS, incubated overnight at 4 °C. After blocking (5% milk in PBST), 500 ng, 250 ng, and 50 ng of scFv-pF and scFv-pC were dispensed in the wells and incubated for 2 hours at 37 °C. The plate was incubated with Anti-6x his antibody (1:2000) and goat anti-mouse secondary antibody (1:3000) and developed with 50 µl/well of TMB substrate (KPL) for 10–30 min. The absorbance was measured at 450 nm using a Perkin-Elmer atomic absorption spectrometer.

### Immunohistochemistry

*Post-mortem* brain tissues were obtained from Newcastle Brain Tissue Resource, Institute of Neuroscience, Newcastle University, UK. Fixed midbrain tissue from a PD case, a DLB case and two aged controls were stained with scFv-pF and -pC. Following dewaxing and epitope unmasking using citrate pH 6 and formic acid, sections were incubated in scFvs (both diluted 1:2 in PBS) for one hour at room temperature. To identify scFv binding, we used anti-6xHis antibody (ab18184, Abcam, Cambridge, UK; 0.5 mg/ml) for one hour at room temperature. Primary antibody binding was then visualized with Menarini MenaPath kits (Menarini Diagnostics, UK) according to manufacturer’s instructions, and utilizing the MenaPath Purple chromagen to enable differentiation of antibody binding compared to neuromelanin, and counterstained with haematoxylin. Donors or next of kin provided informed consent to donate tissues. All procedures were approved according to the Newcastle Brain Tissue Resource Ethics Committee and the local UK National Health Service Research Ethics Committee. All methods involving human *post-mortem* brain tissues were performed based on the guidelines of Newcastle Brain Tissue Resource Ethics Committee and the local UK National Health Service Research Ethics Committee.

### Thioflavin-S (Th-S) aggregation assay

α-syn seeds were incubated in PBS either alone (1 µM), or in combination with scFv-pF (40 µM), scFv-pC (8 µM) or Syn-F2 (1 µM), in sealed 1.5 mL sterile polypropylene tubes for 1 hour at 37 °C with continuous shaking at 300 rpm in a total volume of 100 µL. 25 µM α-syn monomers were added in PBS at a final volume of 500 µL, and the reaction mixture was incubated at 37 °C with continuous shaking at 300 rpm. Aliquots were collected at 1 hour intervals between 0–6 hours and were flash frozen using liquid nitrogen. The aggregation of α-syn was assessed using Th-S fluorescence assay. 10 µL of each sample was mixed with 40 µL of Th-S reagent (25 µM) in PBS. Fluorescence was measured in a 384-well, non-treated, black micro-well plate (Nunc) using a Perkin Elmer EnVision Multimode Plate Reader with excitation and emission wavelengths of 450 nm and 486 nm, respectively.

### Transmission electron microscopy

The samples (5ul) were deposited on Formvar-coated 400-mesh copper grids (Agar Scientific, UK), fixed briefly with 0.5% glutaraldehyde, washed with dd water, negatively stained with 2% uranyl acetate (Sigma) and imaged with a FEI Talos F200C TEM electron microscope at 200 keV.

### Cell culture

Human embryonic kidney (HEK293T) cells and human neuroblastoma SH-SY5Y cells were cultured according to ATCC protocols in culture media at 37 °C in a 5% CO^2^ atmosphere.

### Immunofluorescence

SH-SY5Y or HEK293T cells were plated at a density of 150,000 cells on poly-L-lysine coated coverslips and next day incubated with scFv-pF or -pC at 2 μg/ml concentration for different time-points such as 30 min, 2 hours, 6 hours or 24 hours in Opti-MEM media, washed three times with 1X PBS and fixed in 4% PFA for 20 min, permeabilized with 0.5% Triton-X 100 for 5 min and stained using anti-His antibody (1:500, Abcam), Phalloidin-594 (1:100, Thermo Scientific), and anti-Ms-Alexa-488 (Thermo Scientific). Stained coverslips were visualized using 63X objective with Carl Zeiss wide field upright microscope or confocal microscope. Final images were assembled using Adobe Photoshop software.

### MTT and LDH activity assay

SH-SY5Y cells were plated at a density of 15,000 cells/well in 96-well plate (Nunc), and incubated for 24 hours. 2 µM α-syn pure seeds were incubated alone or with either 0.25 µM Syn-F2, 0.25 µM scFv-pF, or 0.25 µM scFv-pC in 100 µL Opti-MEM for 30 min at 37 °C at 300 rpm shaking. The media was replaced with the 100 µL Opti-MEM containing desired seeds or seeds plus antibodies in two sets. After 1 hour, 10 µM α-syn monomers were added in another 100 µL Opti-MEM to the cells in one set or only 100 µL Opti-MEM was added, and incubated for 48 hours. Control wells consisted of Opti-MEM only with no treatment, and blank wells consisted of no cells. For LDH activity assay 50 μl media was removed before the addition of MTT reagent. 20 µL of 6 mg/mL MTT in PBS was added to each well, and the plate was incubated at 37 °C for 4.5 hours. The medium-MTT solution was removed and 100 µL/well of 15% (w/v) SDS, 50% (v/v) N,N-dimethylformamide, pH 4.7, was added and the plate was incubated overnight at 37 °C. The absorbance was measured at 590 nm using Nano Quant by TECAN. LDH activity was spectrophotometrically measured at 490–620 nm using a Pierce LDH Cytotoxicity Assay kit (Thermo scientific) according to the manufacturer’s indications using 50 μl media from treated cells. LDH activity from total cells lysates with media was defined as the sum of intracellular and extracellular LDH activity, normalized as 100% and then, the amount of LDH released to the extracellular medium was expressed as percentage of this total value in all the other samples.

### Detection of insoluble pS129-α-syn in HEK293T cell model of PD

HEK293T cells in cDMEM were plated at a density of 800,000 cells (2 mL/well) in a 6-well plate, and incubated for 24 hours. The cells were transfected with 2 µg/well of wild-type α-syn plasmid in Opti-MEM using lipofectamine 3000 transfection reagent (Invitrogen) according to manufacturer’s protocol. Four hours after incubation with the transfection mixture, complete DMEM was added to the cells and incubated for 24 hours. The cells were transfected with 0.2 µM pure α-syn seeds similarly and after 4 hours, the cells were either treated with 0.25 µM Syn-F2, 0.25 µM scFv-pF or 0.25 µM scFv-pC for 48 hours. The control wells consisted of Opti-MEM only with no treatment. After 48 hours, triton X-100 soluble (soluble fraction) and SDS-soluble (insoluble fraction) lysates were prepared and quantified using Thermo-Scientific Micro-BCA Protein Assay Kit for equal loading on SDS-PAGE. Western-blotting was performed to detect the phosphorylated α-syn using anti-α-syn (phospho S129) antibody (ab51253, 1:2000) and the total α-syn using Syn-1 antibody (BD Biosciences, 100 ng/mL). Anti-β-actin antibody (C4) Sc-47778 was used as the loading control. ImageJ was used for quantification.

### Ethical agreements

Donors or next of kin provided informed consent to donate tissue. All procedures were approved and performed according to the local UK National Health Service Research Ethics Committee.

## Supplementary information

Supplementary data.

## Data Availability

Data will be provided upon reasonable requests.
