# Supplementary material for: Fibrillar form of α-synuclein-specific scFv antibody inhibits α-synuclein seeds induced aggregation and toxicity
Source: Sci Rep. 2020 May 18;10:8137. doi: 10.1038/s41598-020-65035-8 (PMC7235225; doi:10.1038/s41598-020-65035-8)

Fibrillar form of α-synuclein-specific scFv antibody inhibits α-synuclein seeds induced aggregation and toxicity

Vijay Gupta[1#], Safa Salim[1#], Issam Hmila[1#], Nishant N. Vaikath[1#], Indulekha P. Sudhakaran[1], Simona Ghanem[1], Nour K. Majbour[1], Sara A. Abdulla[1], Mohamed M. Emara[4], Houari B. Abdesselem[1], Tamas Lukacsovich[3], Daniel Erskine[2], Omar M. A. El-Agnaf [1*]

1 Neurological Disorder Research Center, Qatar Biomedical Research Institute (QBRI), Hamad Bin Khalifa University (HBKU), Qatar Foundation, Doha, Qatar.

2 Translational and Clinical Research Institute, Newcastle University, UK.

3 Brain Research Institute, University of Zürich, Switzerland.

4 Basic Medical Sciences Department, College of Medicine, QU Health, Qatar University, Doha, Qatar.

***Corresponding author:** Omar El-Agnaf, Qatar Biomedical Research Institute, Hamad Bin Khalifa University, P.O. Box 34110, Doha, Qatar. Tel: +97455935568; Fax: +974 445 41770;
E-mail: oelagnaf@qf.org.qa

# denotes equal authorship.

a

b

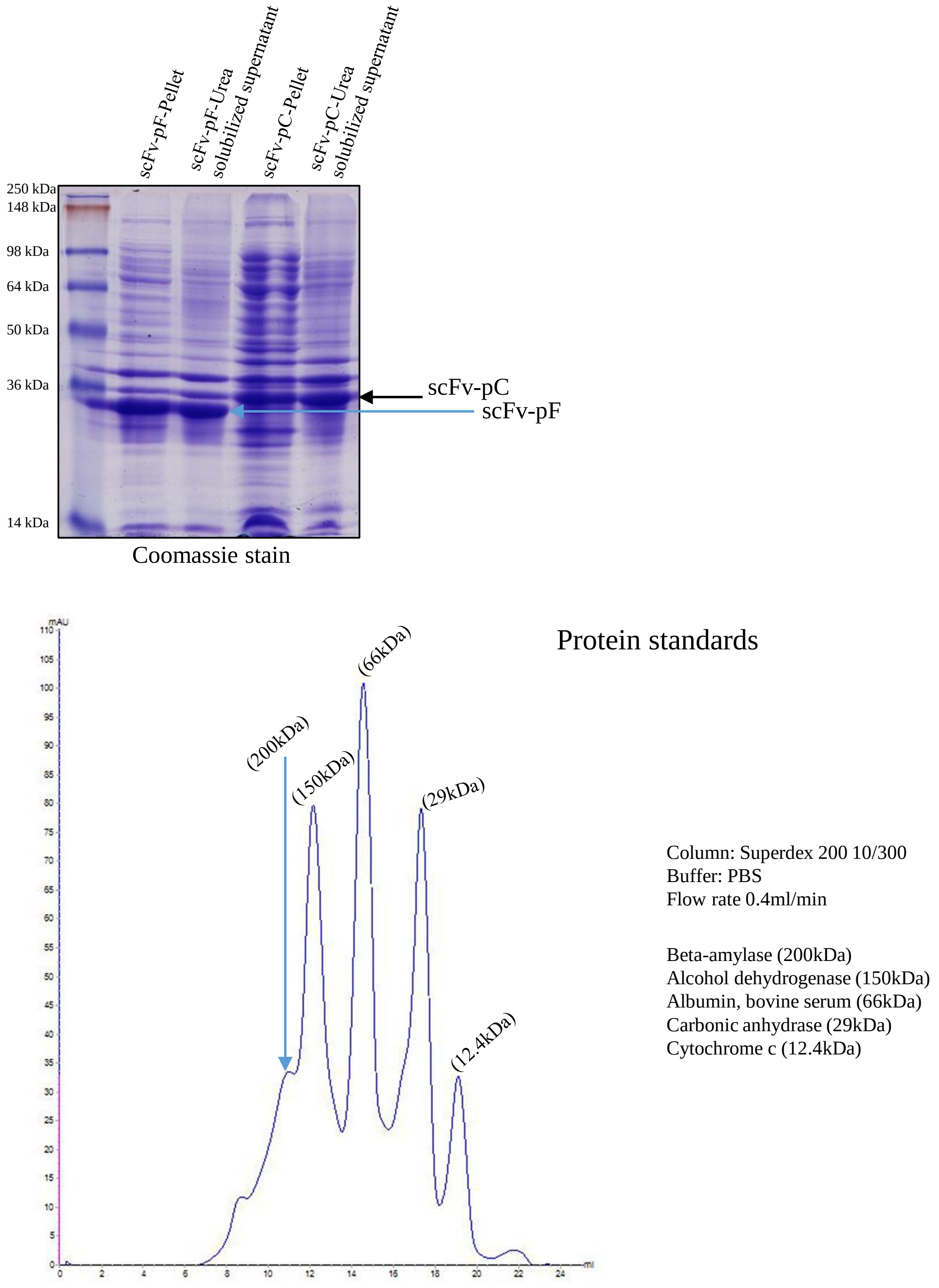

Suppl. Fig. S1. Protein yield of scFv-pF and scFv-pC fusion proteins during urea solubilization and size exclusion chromatography. (a) Inclusion body pellets (suspended in 50 ml -1x PBS per liter) from scFv-pF and scFv-pC protein's expression were washed with 1M urea + 1% Triton-X 100 solution five times and then with 1X PBS solution and solubilized in 2M urea, pH-12.5 solution for 2-3 hours (25 ml per liter). SDS-PAGE analysis of expression of scFv-pF and -pC in inclusion body pellet after E.coli lysis (inclusion body pellet) is compared with 2M urea, pH-12.5 solubilization in equal amounts. scFv-pF and -pC are indicated by an arrow and show around 50% recovery. (b) Size exclusion chromatography showing the control run wherein Beta-amylase (200kDa), Alcohol dehydrogenase (150kDa), Albumin, bovine serum (66kDa), Carbonic anhydrase (29kDa) and Cytochrome c (12.4kDa) were used as protein standard controls.

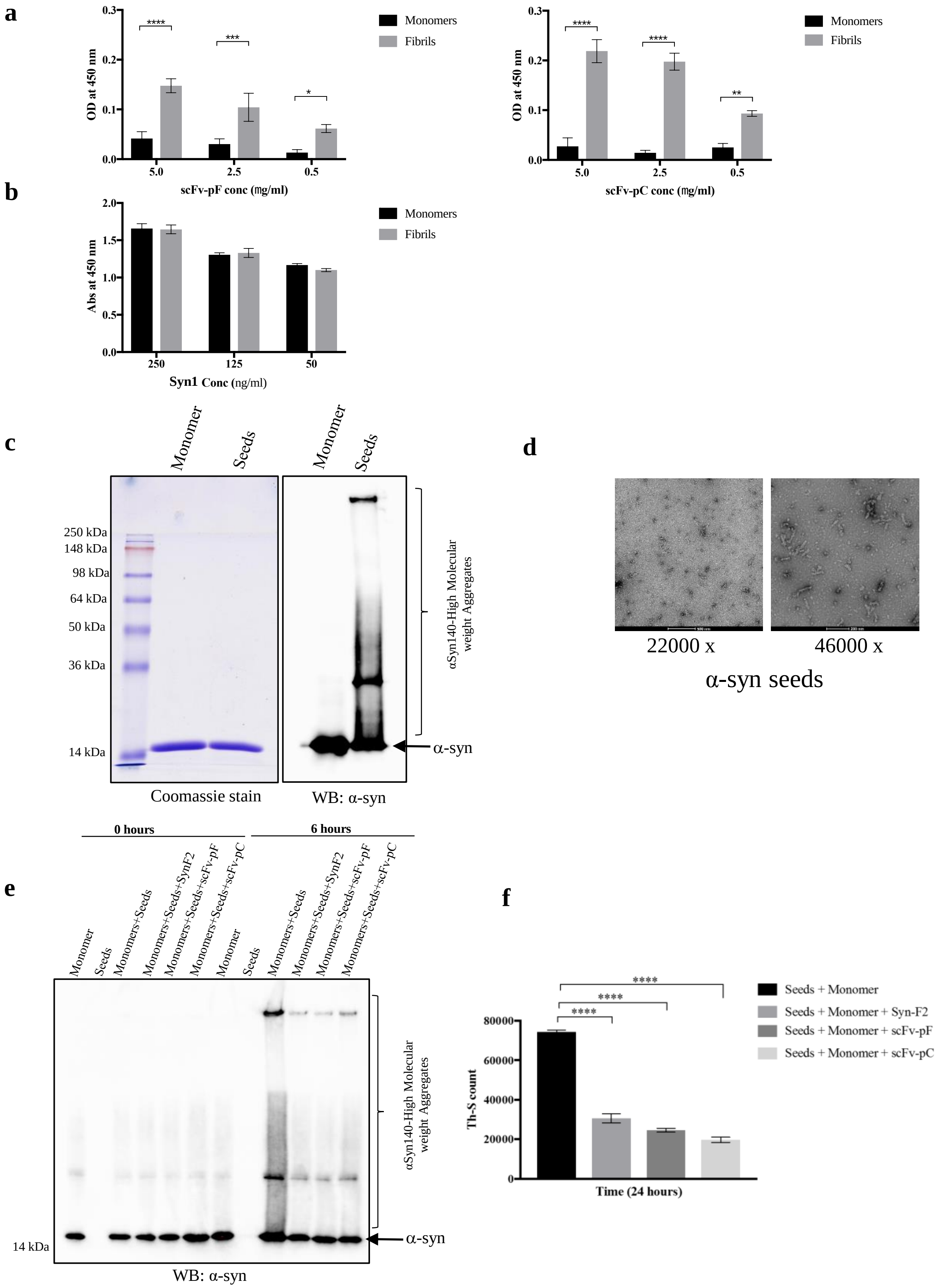

Suppl. Fig. S2. Characterization of scFvs and their effect on seeded aggregation. ELISA showing specific binding of scFv-pF and -pC to fibrillar form of α-syn. 100ng of α-syn monomers or fibrils were coated on a 96-well MaxiSorp plate and indirect ELISA was performed using indicated concentrations of scFv-pF/-pC(a) or Syn-1(b). Statistical analysis was performed using one-way ANOVA with Dunnet's multiple comparison test. (***, $p < 0.001$; **, $p < 0.01$; *, $p < 0.05$). (c) SDS-PAGE analysis of 10 µg α-syn monomers and seeds in denaturing sample buffer with SDS and boiling (left panel) show a single band and did not show the presence of any impurity. Right panel show western blotting of 100 ng α-syn monomers and seeds in non-denaturing sample buffer (without any boiling and SDS) using α-syn specific antibody (FL140, Santa Cruz) as shown earlier[39]. The presence of high molecular weight aggregates and insoluble aggregates that do not enter the stacking gel is clearly visible in seeds. (d) Electron microscopy images of negatively stained α-syn seeds (70µM) show their uniformity and small size in the range of ~50-200nM. Scale bar = 500nm and 200nm. (e) 1µl protein samples at 0 hours and 6 hours time-points from the experiments shown in Fig. 2b were prepared in non-denaturing sample buffer (without any boiling and SDS) and loaded on SDS-PAGE and western blotting was performed using α-syn specific antibody (FL140, Santa Cruz). (f) *In vitro* seeding of α-syn aggregation showing inhibition by Syn-F2, scFv-pF and -pC at 24 hours as described in Fig. 2b.

a

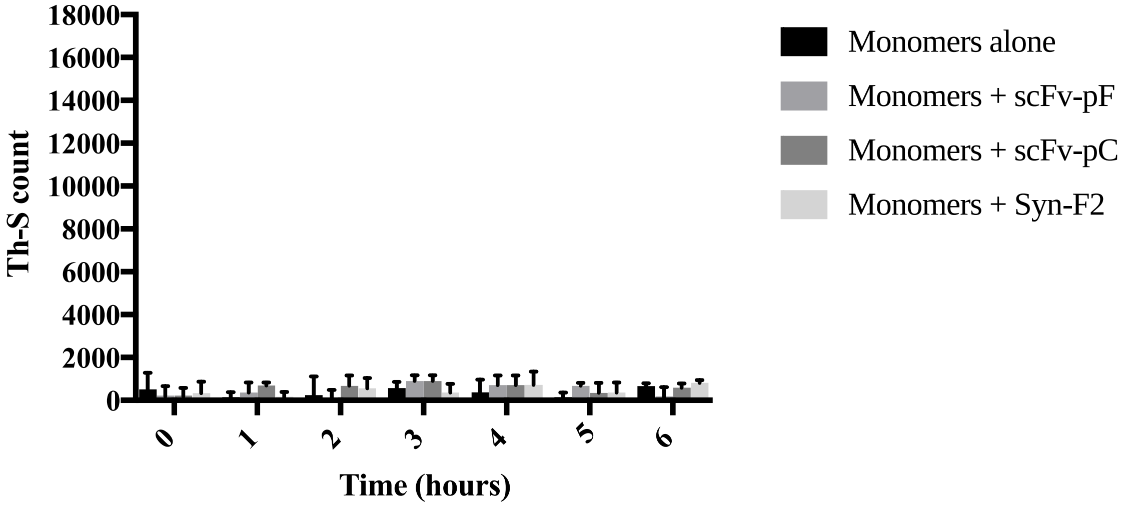

b

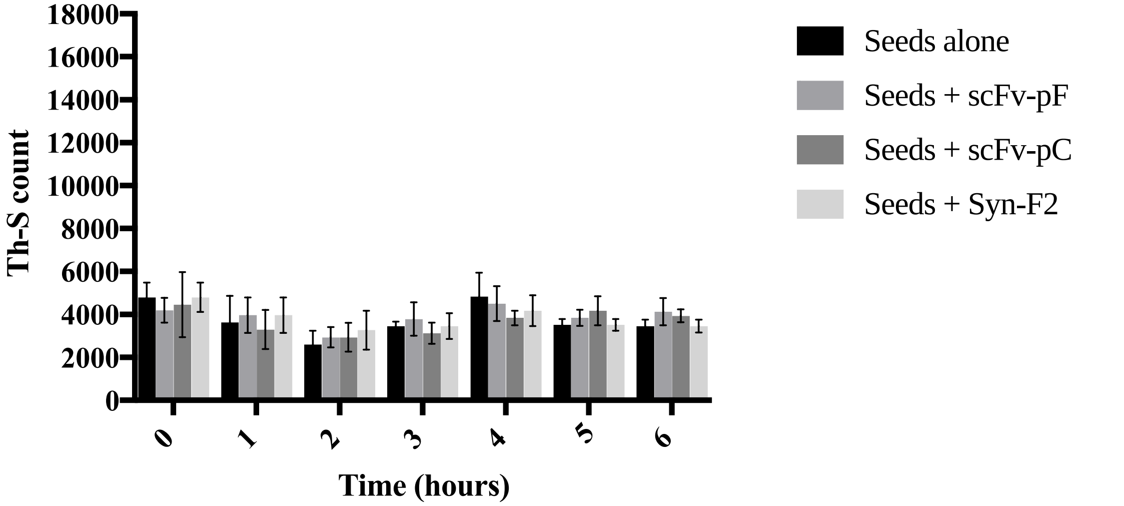

Suppl. Fig. S3. Th-S fluorescence assay showing the extent of fibrillation in α-syn monomers or seeds alone or with Syn-F2 and scFv-pF/-pC. α-syn monomers (25μM) (a) or seeds (1μM) (b) were incubated alone or with Syn-F2 (1μM), scFv-pF (40 μM) and scFv-pC (8 μM). The extent of fibrillation was estimated by the Th-S fluorescence assay at indicated time-points. α-syn monomers showed negligible counts up to six hours which did not increase by incubating with Syn-F2 or scFv-pF/-pC (a). On the other hand, α-syn seeds had a basal Th-S count of 4000-6000 which did not change with increasing time or by incubating with Syn-F2 or scFv-pF/-pC (b). The assay was performed in triplicate (average of triplicate measurements ± standard deviations).

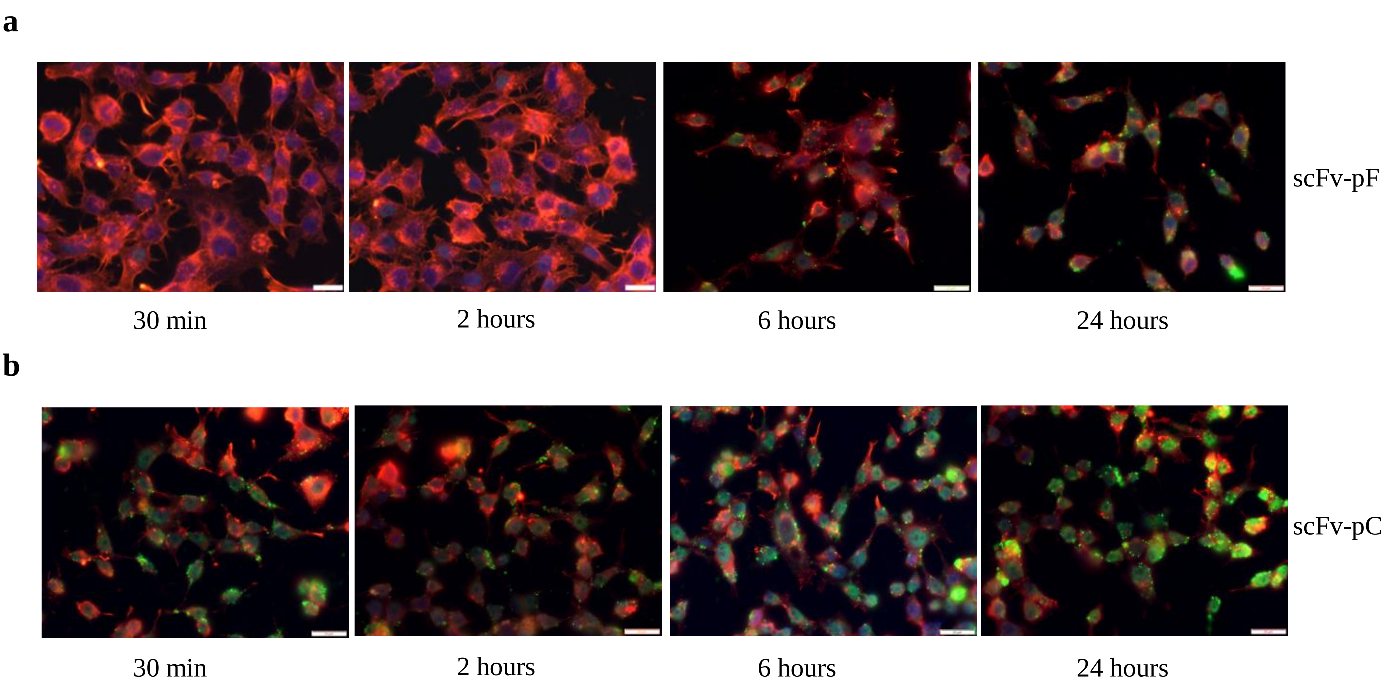

Suppl. Fig. S4. Localization of scFv-pF/pC upon incubating with SH-SY5Y cells at different time-points. SH-SY5Y cells were incubated with scFv-pF and scFv-pC in Opti-MEM at 2µg/ml for 30min, 2hours, 6 hours and 24 hours and fixed and stained using anti-His antibody and Phalloidin-594 and imaged using wide-field microscope. scFv-pF did not show any specific staining pattern at initial time-points of 30min and 2 hours, however at later time points of 6 hours and 24 hours it started showing cell membrane binding whereas on the other hand scFv-pC showed specific staining pattern around the cells at all time-points indicating efficient membrane binding and intracellular delivery. Scale bars = 10µm.

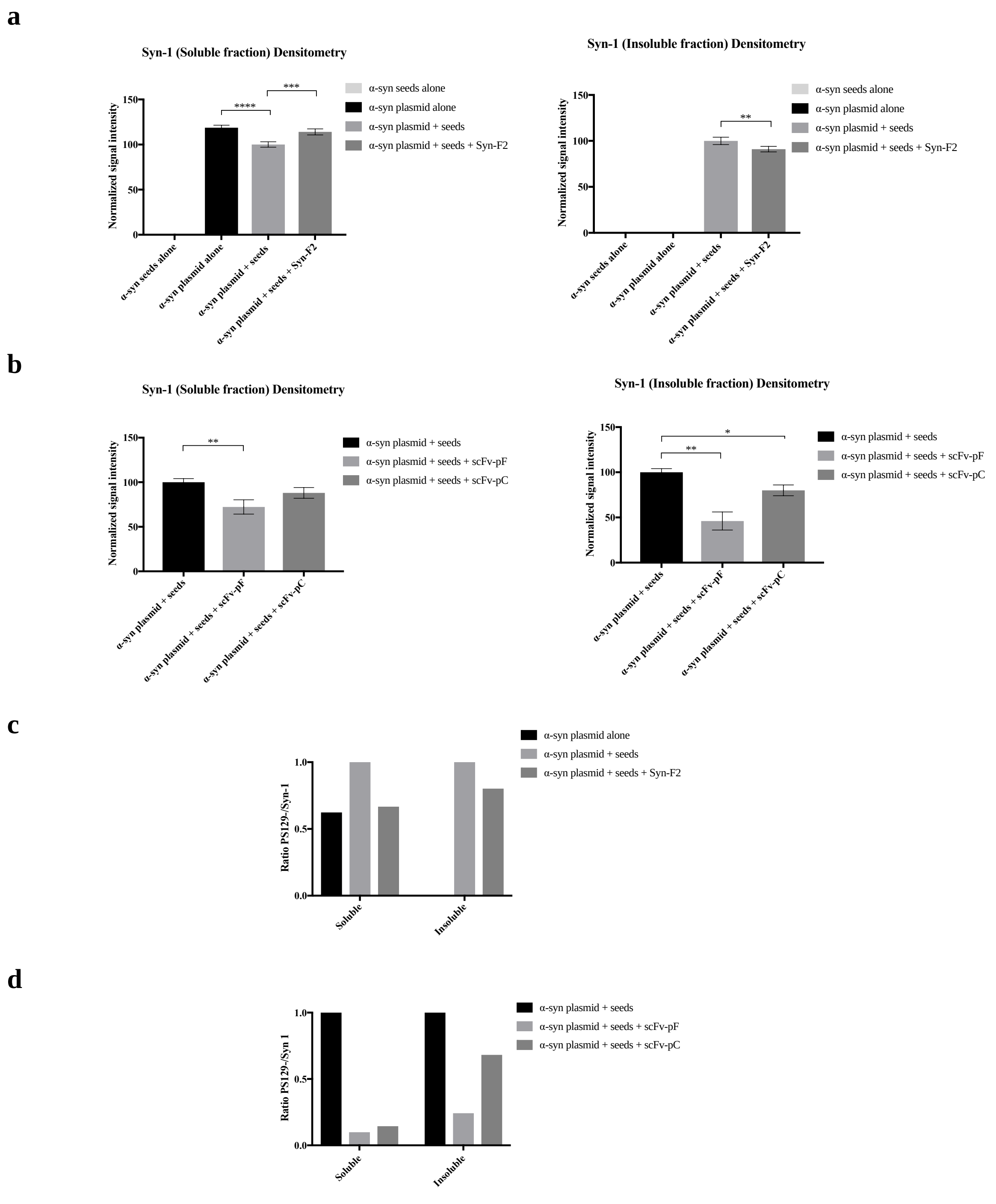

Suppl. Fig. S5. Syn-F2, scFv-pF and scFv-pC decrease aggregated α-syn in HEK293T cell model of PD. Western blotting data from Fig. 5 was quantified using densitometric analysis and Syn-1 protein levels were plotted from Fig. 5a in (panel a) and Fig. 5b in (panel b). Ratios of pSer129 to Syn-1 were plotted from Fig. 5a in (panel c) and Fig. 5b in (panel d). Statistical analysis was performed using one-way ANNOVA with Dunnet's multiple comparison test. ((****, $p < 0.0001$, ***, $p < 0.001$; **, $p < 0.01$).

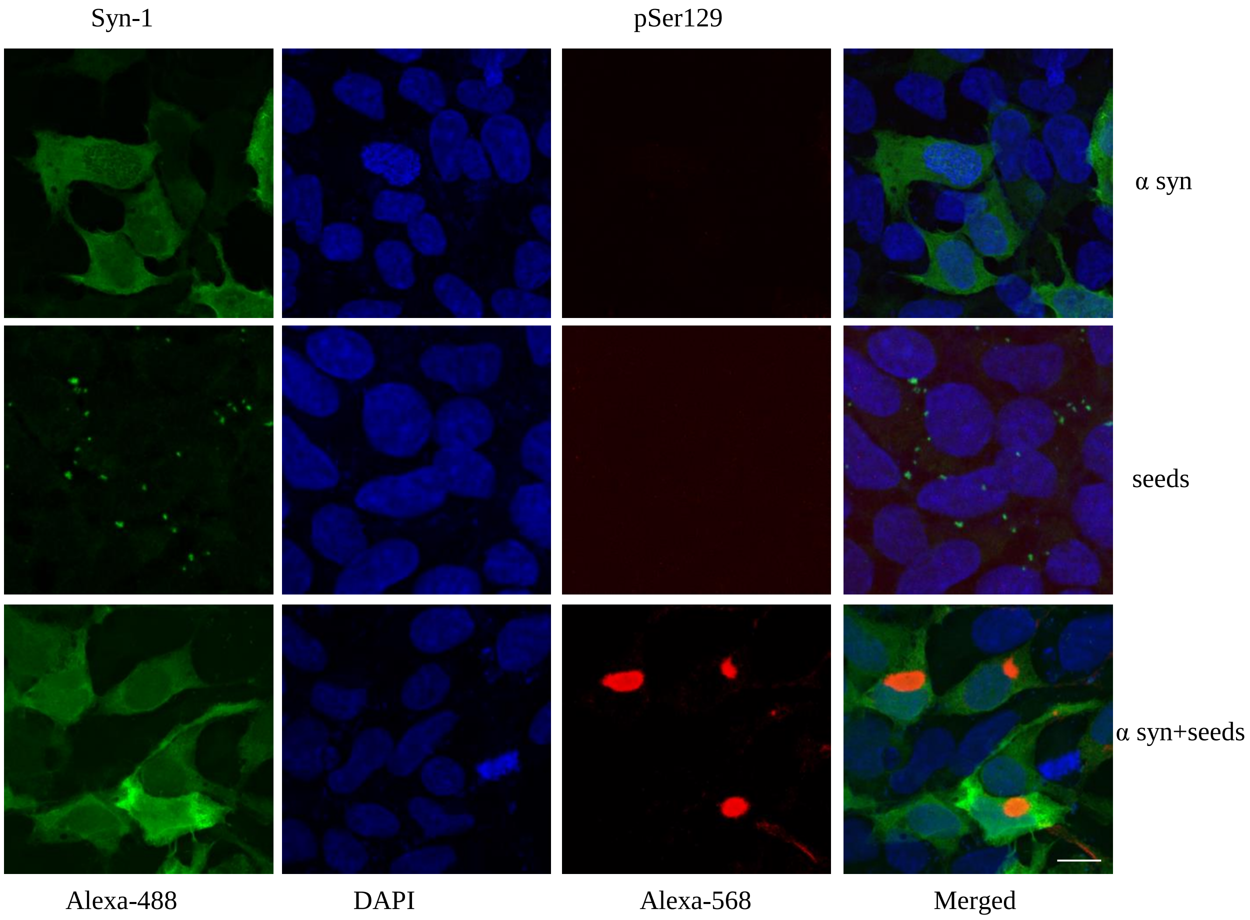

Suppl. Fig. S6. Immunocytochemistry images showing the formation of α-syn aggregates in HEK293T cells. HEK293T cells were transfected with wild type α-syn plasmid and similarly with 0.2µM α-syn seeds the following day and then incubated for next 48 hours. As controls one group of cells was either transfected with α-syn plasmid or α-syn seeds (0.2µM). Cells were fixed and co-stained using anti-pSer129 antibody (Alexa-568) and α-syn specific antibody (Syn-1) (Alexa-488) and imaged using confocal microscope. Serial optical sections in the *Z*-axis of the cell, collected at 1 µm intervals with 63× oil immersion objective lense (NA 1.4) were projected and observed in a total thickness of 10 µm by using LSM 780 (version 3.2) software. Cells transfected with α-syn plasmid shows diffused cytosolic expression and seeds show small aggregates inside and around cells. However, upon co-incubation of α-syn plasmid with α-syn seeds, bigger aggregate formation is detected by pSer129 antibody. α-syn plasmid or α-syn alone do not show pSer129 antibody staining indicating the absence of phosphorylated aggregated forms. Scale bars = 10µm.

Amino acid sequence of scFv-pF

MMSPAQFLFLLVLWIQETNGDVVMTQTPLTLSVTIGQPASISCKSSQSLLYSNGKTYLNWLLQ
RPGQSPKRLIYLVSKLDSGVPDRFTGSGSGTDFTLKISRVEAEDLGVYYCVQGTHFPTFGGGT
KLEIK**GGGGSGGGGSGGGGS**MDSRLNLVFLVLILKGVQCDVQLVESGGGLVQPGGSRKLSC
AASGFTFSSFGMHWVRQAPEKGLEWVAYISSGSSTIYYADTVKGRFTISRDNPKNTLFLQMTS
LRSEDTAMYYCARGNNPGTGYYYAMDYWGQGTSVTVSSLVPRGSLE**HHHHHH**

Amino acid sequence of scFv-pC

RQIKIWFQNRRMKWKKMMSPAQFLFLLVLWIQETNGDVVMTQTPLTLSVTIGQPASISC
KSSQSLLYSNGKTYLNWLLQRPGQSPKRLIYLVSKLDSGVPDRFTGSGSGTDFTLKISRVEA
EDLGVYYCVQGTHFPTFGGGTKLEIK**GGGGSGGGGSGGGGS**MDSRLNLVFLVLILKGVQ
CDVQLVESGGGLVQPGGSRKLSCAASGFTFSSFGMHWVRQAPEKGLEWVAYISSGSSTIYY
ADTVKGRFTISRDNPKNTLFLQMTSLRSEDTAMYYCARGNNPGTGYYYAMDYWGQGTS
VTVSSLVPRGSLE**HHHHHH**

Suppl. Fig. S7. Amino acid sequences translated from the coding sequence of scFv-pF and scFv-pC is presented. Cell penetrating peptide-16 amino acid long, $(Gly_4Ser)_3$ and 6X-His tag is in bold letters.

Manuscript Figure 5 B: Soluble Fraction: Full Blot scan

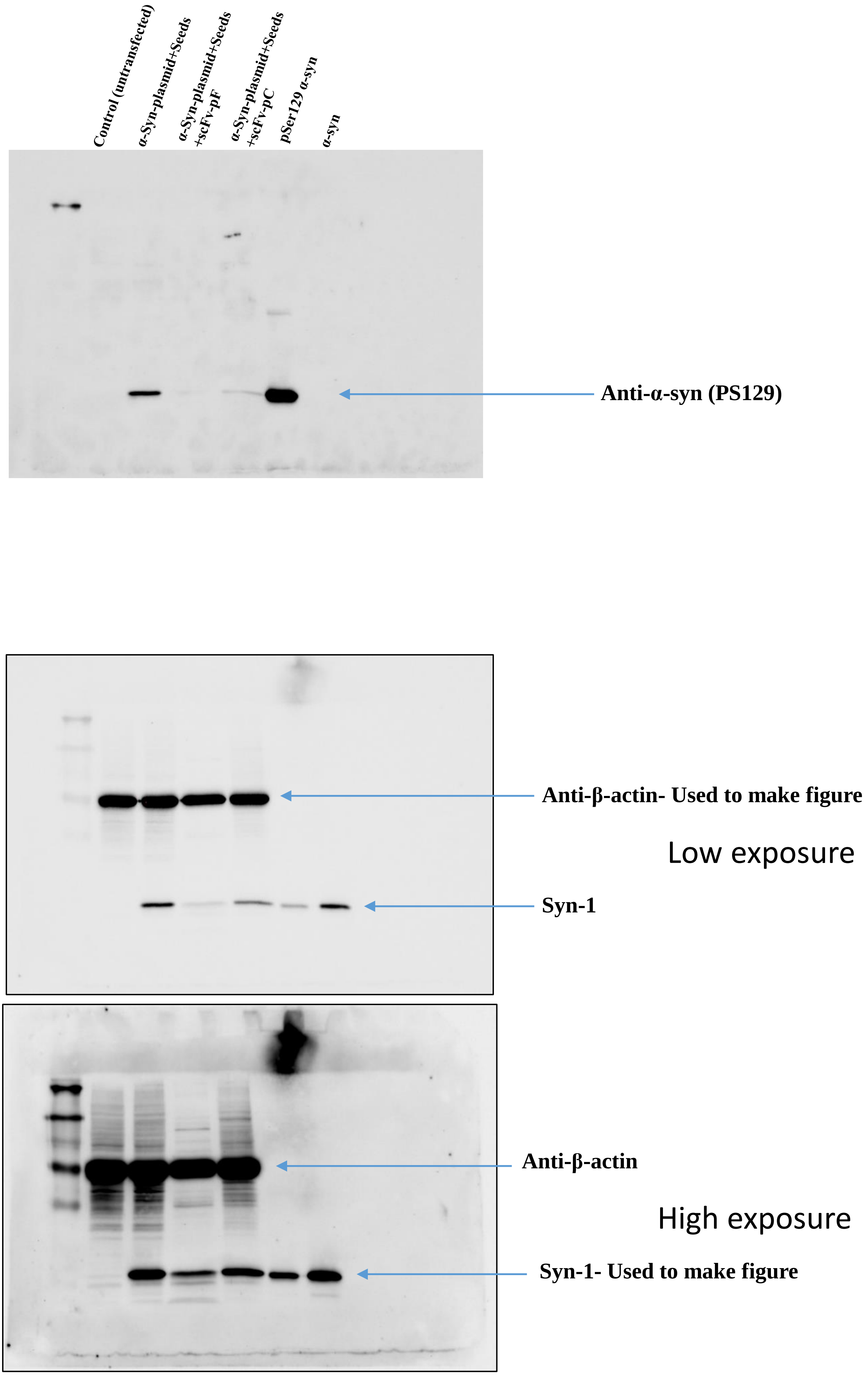

Manuscript Figure 5 B: Insoluble Fraction: Full Blot scan

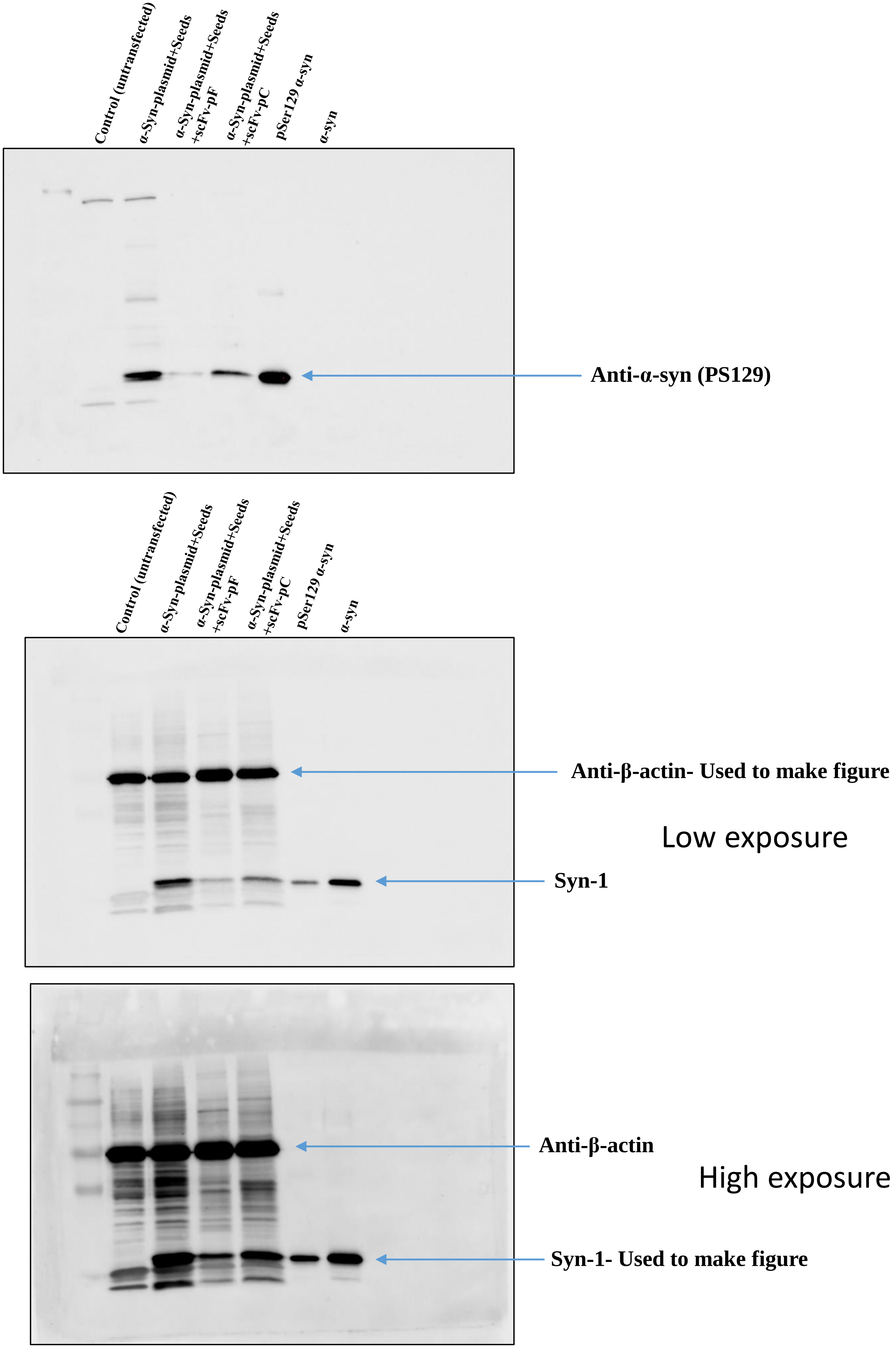

Manuscript Figure 5 A: Soluble Fraction: Full Blot scan

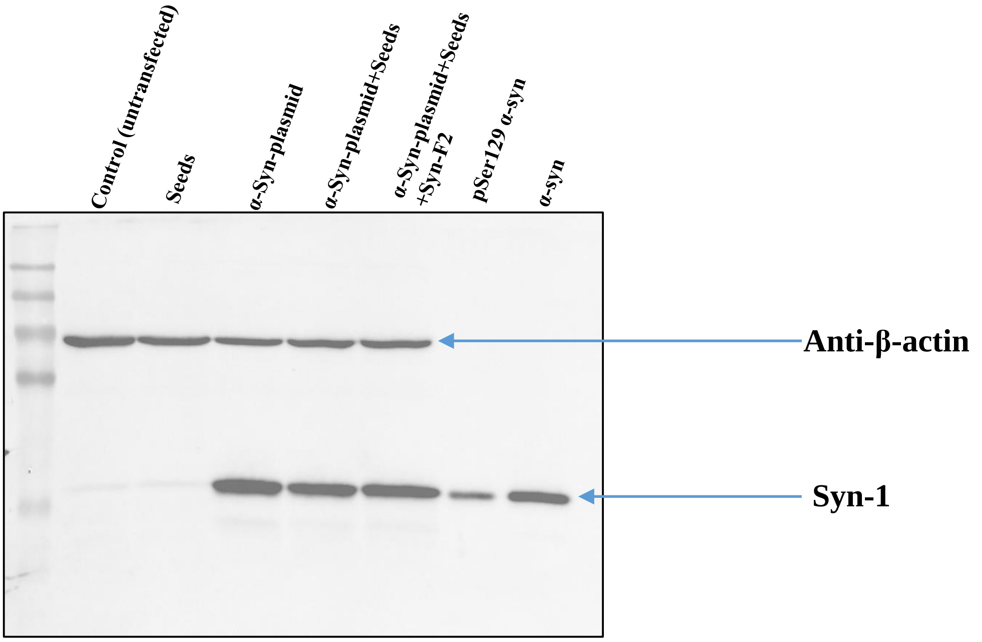

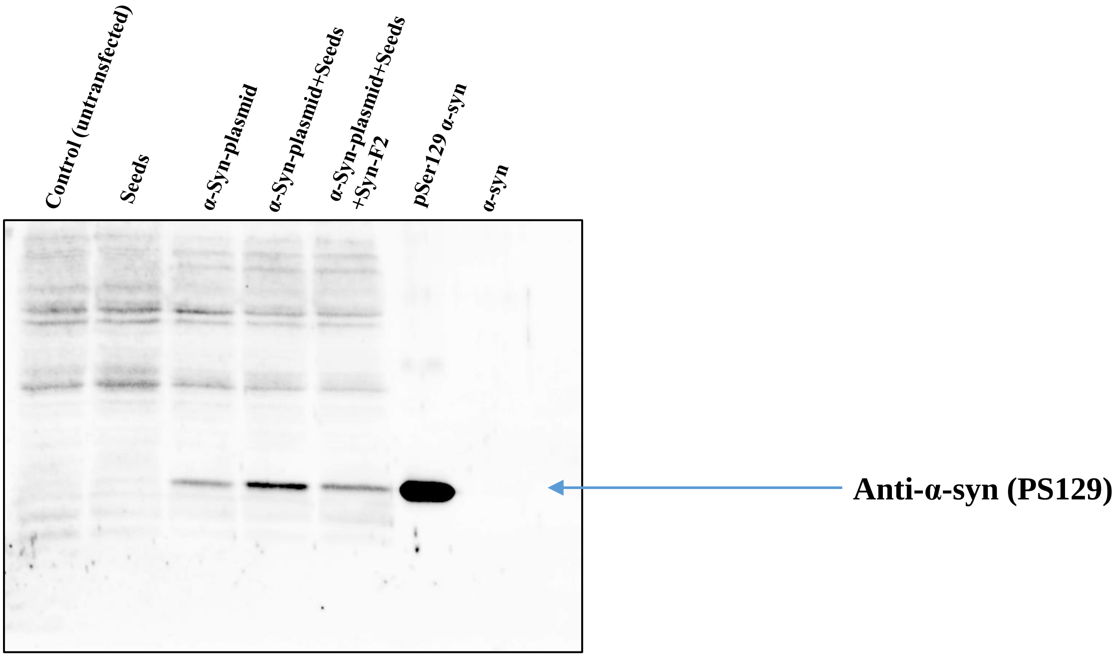

Manuscript Figure 5 A: Insoluble Fraction: Full Blot scan

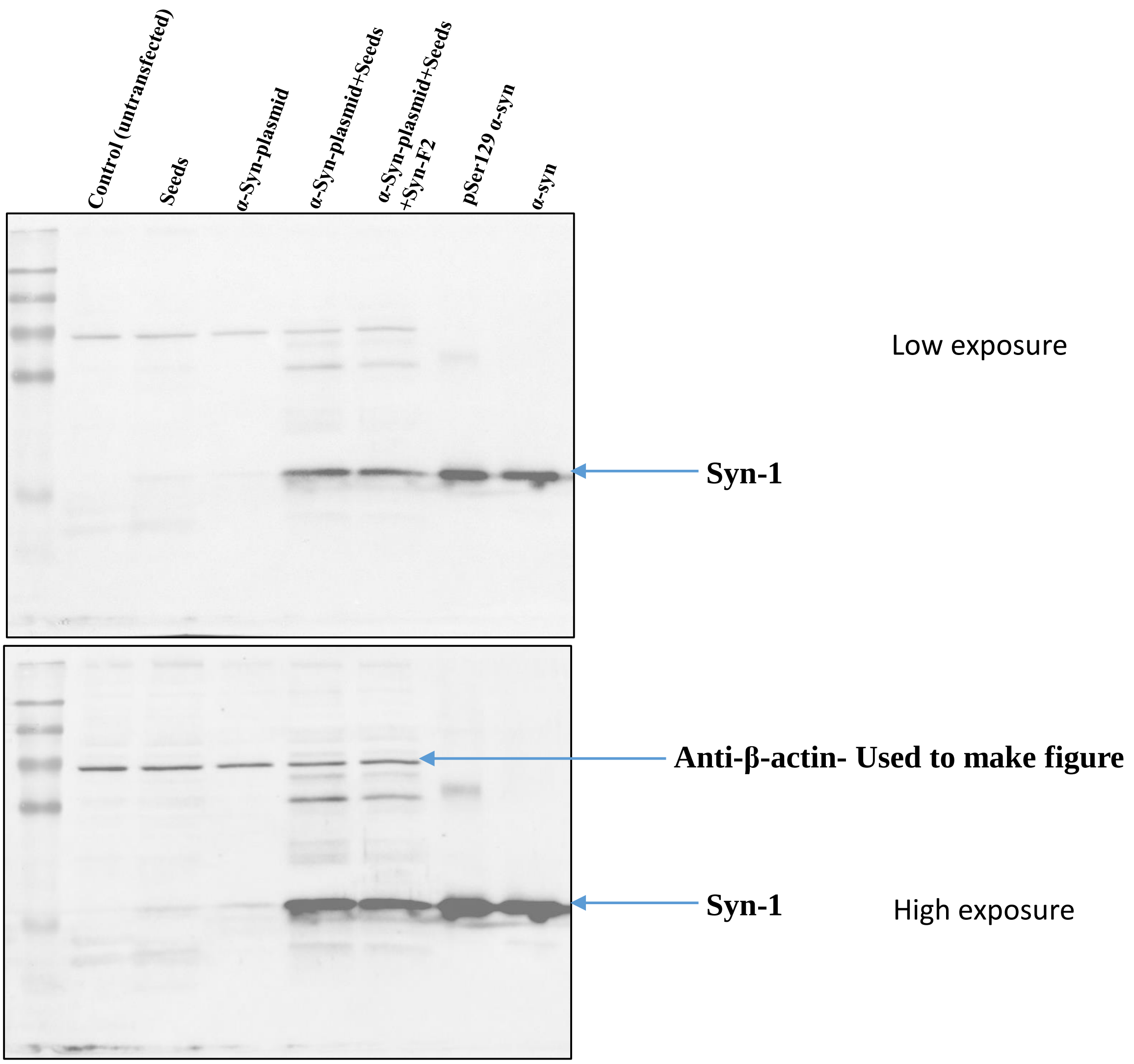

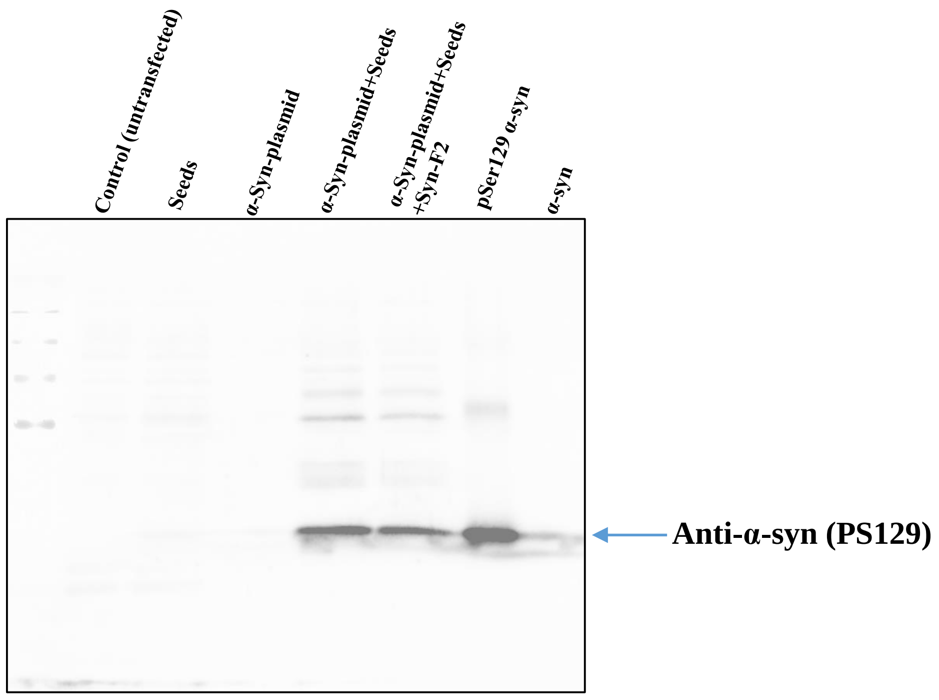

Supplement: Supplementary file 1 — Supplementary data. [file 41598_2020_65035_MOESM1_ESM.pdf]
